# Elevated expression of wildtype *RhoC* promotes* ErbB2-* and* Pik3ca-*induced mammary tumor formation

**DOI:** 10.1186/s13058-024-01842-5

**Published:** 2024-05-28

**Authors:** Nandini Raghuram, E. Idil Temel, Toshihiro Kawamata, Katelyn J. Kozma, Amanda J. Loch, Wei Wang, Jessica R. Adams, William J. Muller, Sean E. Egan

**Affiliations:** 1 Program in Cell Biology, The Peter Gilgan Centre for Research and Learning, The Hospital for Sick Children, 686 Bay Street, Room 16-9703, Toronto, ON M5G 0A4 Canada; 2 Department of Molecular Genetics, The University of Toronto, Toronto, ON M5S 1A8 Canada; 3 Department of Biochemistry and Department of Medicine, Rosalind and Morris Goodman Cancer Research Institute, McGill University, Montreal, QC H3A 1A3 Canada

## Abstract

Copy number gains in genes coding for Rho activating exchange factors as well as losses affecting genes coding for RhoGAP proteins are common in breast cancer (BC), suggesting that elevated Rho signaling may play an important role. Extra copies and overexpression of *RHOC* also occur, although a role for RhoC overexpression in driving tumor formation has not been assessed in vivo. To this end, we report on the development of a Rosa26 (R26)-targeted *Cre-*conditional *RhoC* overexpression mouse (R26^*RhoC*^). This mouse was crossed to two models for *ERBB2/NEU*^+^ breast cancer: one based on expression of an oncogenic *ErbB2/Neu* cDNA downstream of the endogenous *ErbB2* promoter (*FloxNeoNeu*^*NT*^), the other, a metastatic model that is based on high-level expression from MMTV regulatory elements (*NIC*). RhoC overexpression dramatically enhanced mammary tumor formation in *FloxNeoNeu*^*NT*^ mice but showed a more subtle effect in the NIC line, which forms multiple mammary tumors after a very short latency. RhoC overexpression also enhanced mammary tumor formation in an activated *Pik3ca* model for breast cancer (*Pik3ca*^*H1047R*^*)*. The transforming effect of RhoC was associated with epithelial/mesenchymal transition (EMT) in *ErbB2/Neu*^*NT*^ and *Pik3ca*^*H1047R*^ systems. Thus, our study reveals the importance of elevated wildtype Rho protein expression as a driver of breast tumor formation and highlights the significance of Copy Number Abberations that affect Rho signalling.

**Supplementary Information:**

The online version contains supplementary material available at 10.1186/s13058-024-01842-5.

## Introduction

RHO subfamily GTPases (RhoA, B and C), regulate intracellular signaling pathways, several of which coordinates actin dynamics [1]. Their expression and signaling has been found to be altered in many cancer types [2, 3]. Although RHO subfamily members share high sequence homology and have functional similarities, they play unique roles in the coordination of cell signaling and motility of normal and cancer cells. RhoC in particular has been linked to cell proliferation as well as to migration/invasion [2–4]. The mechanism by which RhoC is regulated and through which it signals in cancer have yet to be defined in detail. Upstream, p53 and Ets transcription factors, as well as microRNAs play an important role in controlling RhoC protein expression. RhoGDI, RhoGAP and RhoGEF proteins control GTP-loading [4–6]. Whereas Rock kinases, Rac and Cdc42, Formin-like proteins, as well as microfilaments and microtubules seem to play important roles downstream [4].

Multiple studies have identified a link between *RhoC* and metastatic dissemination [7–9]. Overexpression of wildtype *RhoC *in vitro drives invasion of HME and MCF10A cells in both 2D and 3D culture [10, 11], and high levels of *RhoC* observed in the SUM149 inflammatory breast cancer cell line are thought to increase production of pro-angiogenic factors [11]. In vivo, knockout of *RhoC* in the Polyoma Virus Middle T mouse model reduces metastatic invasion of mammary tumors [9]. A number of studies have demonstrated that *RHOC* overexpression is common in aggressive BC [12]. For example, some *ERBB2*^+^ and mutant *PIK3CA* breast tumors show elevated *RHOC* expression [13]. Whether increased levels of wildtype RHOC promote tumor formation and/or progression in this context has never been determined. In this study, we describe the development of a novel mouse model for Cre-conditional overexpression of *RhoC*. Furthermore, we describe the use of this mouse to test for cooperation between elevated wildtype *RhoC* expression and activated *ErbB2* or activated *Pik3ca* in transformation of mammary epithelium in vivo.

## Materials and methods

### Mouse colony maintenance and genotyping

All mouse strains used in this study were maintained at the Centre for Phenogenomics in accordance with guidelines established by the Canadian Council on Animal Care (CCAC). Only female virgin mice were studied in mammary tumor experiments. Mice were genotyped with primer sets listed in Additional file 1: Supplementary Table 6.

### Necropsy and tumor collection

Experimental mice were monitored for tumor formation for 18 (540 days) or 24 months (720 days). When mice reached humane endpoint, they were sacrificed according to CACC guidelines. Upon sacrifice, mammary tumors were collected and a portion of each (along with adjacent normal mammary tissue) fixed in 10% phosphate buffered formalin phosphate (Fisher Scientific HC200-20) at room temperature for a minimum of 24 h. The remainder of each tumor was divided into smaller pieces and placed on dry ice or in RNAlater (Qiagen). Samples were placed at − 80 °C for long-term storage.

### Histological analysis and immunohistochemistry

Formalin-fixed tissue samples were paraffin-embedded by the Pathology Core at the Centre for Modeling Human Disease (CMHD) in The Centre for Phenogenomics. 5 μm sections were stained with Hematoxylin and Eosin and used for histological analysis. Also, sections were used for staining by IHC as previously described [14].

### Statistical analysis of mammary tumor-free survival

All statistical analysis was performed in R (http://www.r-project.org/) and GraphPad Prism (version 7.0). Mammary-tumor free survival was modeled using Kaplan–Meier curves. Curves were generated using ‘survival’ library and ‘survfit’ functions. Survival statistics were calculated as non-parametric log rank p-values for censored data using the ‘survdiff’ function. In each experiment, mice that reached endpoint due to conditions unrelated to mammary tumor development (typically either lymphoma or thymoma) were censored. T-tests and proportion tests were calculated using the standard and ‘plotrix’ libraries in R. Significant statistical difference was defined as *p* < 0.05 and t-tests were run two-sided at a 95% confidence interval.

### Generation of a Cre-inducible ROSA26-RhoC-IRES-eGFP overexpression mouseline

To clone mouse RhoC, a pCMV-Sport6-RhoC plasmid was obtained from The Centre for Applied Genomics at the Hospital for Sick Children. 100 ng of template plasmid DNA was then used to PCR amplify RhoC modified through the addition of 5′ EcoRI and NheI restriction sites (forward primer: 5′- GAATTC GCTAGC-TCAGCCATGGCTGCGATCCGAAAG -3′) and a 3′ EagI restriction site (5′- CGGCCG-TCAGAGAATGGGACAGCCCCTCCG -3′). IRES-eGFP was amplified from the pBTG vector (forward primer: 5′- CGGCCG GCCCCTCTCCCTCCCCCCCC -3′ and reverse primer: 5′- CTCGAG TTACTTGTACAGCTCGTCCATGCCG -3′) and flanked by 5′ EagI and 3’ XhoI sites. Both fragments were cloned into TOPO2.1 (TA cloning kit, ThermoFisher Scientific, K204001) and confirmed by sequencing. RhoC and IRES.eGFP were then subcloned together into pcDNA3.1. Finally, a RhoC-IRES-eGFP DNA insert was subcloned into the pBigT shuttle vector and subsequently into pRosa26Pam1. R1 mESC cells were electroporated with the linearized targeting vector (pRosa26Pam1-RhoC-IRES-eGFP) and put under G418 selection for 7 days. Resistant colonies were individually picked into 96-well plates and expanded for DNA analysis, chromosome counting, and storage at − 80 °C. Genomic DNA extractions (DNeasy Blood and Tissue Kit, Qiagen, 69506) were performed for each mESC clone and used to determine correct targeting at Rosa26 by 5′ junction PCRs. Only correctly targeted diploid clones were functionally assessed. These were submitted for morula aggregation at the Transgenic Core in The Centre for Phenogenomics, and resulting high percentage chimeras bred with FVB to obtain germline transmission.

### Transient transfection

T47D human breast cancer cells were plated in 100 mm cell culture dishes and cultured for 24 h before transfection with a pEGFP-C2-based GFP-RhoC construct (Addgene #23226) [15] carrying wild-type human RhoC sequence. An EGFP (Addgene # 6083-1) control plasmid was transfected into parallel cultures. In each case, transfection was performed using Lipofectamine 2000 as per manufacturer’s instructions. GFP expression was observed under a fluorescence microscope at 24 and 48 h following transfection and cells were collected after the second imaging for protein extraction and western blot analysis.

### Western blot analysis

Transfected T47D cells were lysed in 1 × RIPA buffer supplemented with protease inhibitors (RIPA Lysis Buffer System, Santa Cruz SC-24948A) and lysates cleared of debris by centrifugation at 4 °C. 30-100ug of cell lysates were separated on an SDS-PAGE gel and transferred onto a nitrocellulose membrane (Bio-Rad, 162-0115). Blocking was performed in 5% reconstituted milk powder and washing of blots done according to standard protocols. Membranes were incubated in primary antibody overnight at room temperature and secondary antibody for 1 h, also at room temperature. Antibodies and dilutions used are listed in Additional file 1: Supplementary Table 6. For protein detection, ECL reagents (SuperSignal West Pico, Thermo Scientific 1,856,135) were applied to membranes for 5 min followed by imaging and quantification using ImageLab software (http://www.bio-rad.com/en-ca/product/image-lab-software).

### ddPCR analysis

Digital droplet PCR was performed to determine copy number aberrations (CNA) for activated Neu (NeuNT) using an amplicon-specific probe (5’-ACTGTAGTGGGCGTCC-3’). Mouse Grb7 CNA was detected using a commercially available assay (Thermo Scientific, Catalogue number: Mm00602418_cn).

### Bulk RNA sequencing analysis

RNA was isolated from tumors using a Qiagen RNeasy Kit (Cat # 74104) and samples were sequenced using the Illumina NovaSeq 6000 system (S4 flowcell, PE 2 × 150 bp, 70–100 × coverage) at The Centre for Applied Genomics in the Hospital for Sick Children. The quality of FASTQ data was assessed using FastQC (v 0.11.5). Trim Galore (v 0.5.0), and Cutadapt (v 0.10) software were used to trim adaptors. Trimmed reads were screened for contaminating rRNA and mtRNA using FastQ-Screen (v 0.10.0). The distribution of reads across exonic, intronic, and intergenic sequences was assessed using the RSeQc package (http://rseqc.sourceforge.net/, v.2.6.2). Next, alignment to the reference genome was performed on raw trimmed reads (STAR aligner, v 2.6.0c.). To obtain gene counts, filtered STAR alignments were processed to extract raw read counts for individual genes (htseq-count v.0.6.1p2). Only uniquely mapping reads were counted, with any reads that aligned to more than one gene discarded. MultiQC (v1.9) was used to produce a consolidated report containing data from; trimmed and untrimmed reads screened by FastQC as well as data from RSeQC, FastQ Screen, STAR alignments, and htseq-count. Genes differentially expressed between tumors were identified using DESeq2 (v 1.26.0) and R v 3.6.1 (http://master.bioconductor.org/packages/release/workflows/vignettes/rnaseqGene/inst/doc/rnaseqGene.html).

## Results and discussion

### Copy number-dependent overexpression of RhoC in human breast cancer

**Fig. 1:**
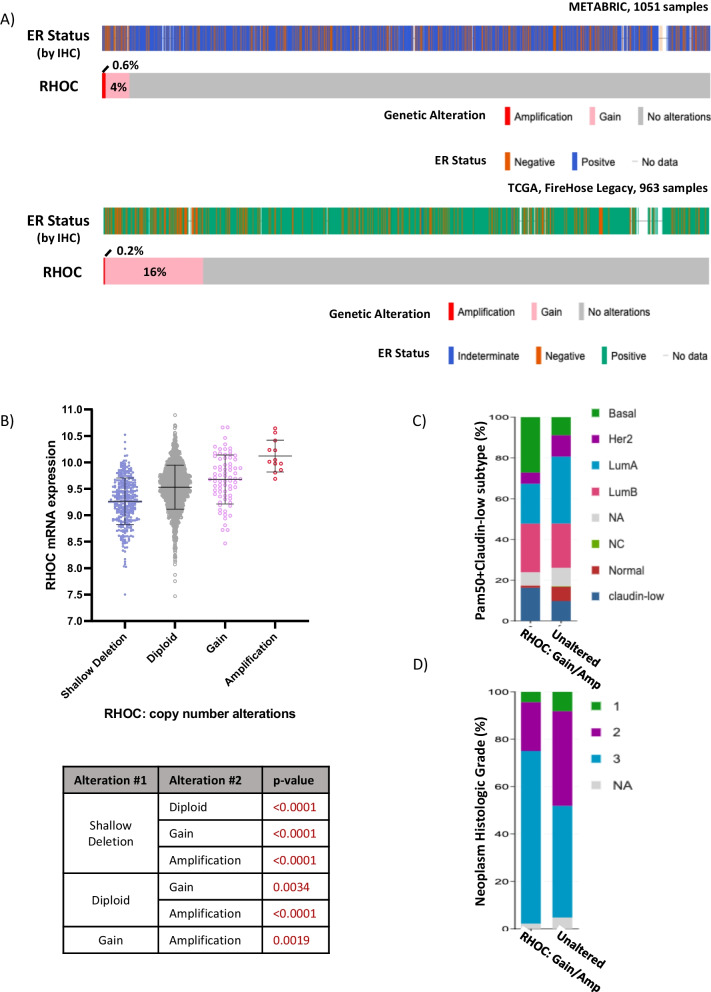
The frequency of *RHOC* copy number gain and amplifications in human breast cancer. **A** *RHOC* gains occur in 4% (top) and 16% (bottom) of human breast tumors from METABRIC and TCGA studies, respectively. The ER status of each breast tumor sample is shown. **B** *RhoC* Copy number and its association with mRNA expression for this gene—all comparisons in the table below are statistically significant. **C, D** Breast tumor subtypes (**C**), and tumor grades (**D**) are displayed for each group—samples with RHOC gain/amplification vs. those without these alterations. All data are from the METABRIC study

Breast tumor formation and progression are associated with copy number aberrations, single-nucleotide variants and other indels as well as with structural variants. Many of the copy number changes affect Rho signaling [5]. For example, the DLC1 RhoGAP on chromosome 8p, shows hemizygous deletion in 40 to 50% of breast tumors, and homozygous deletion occurs in a small fraction of cases (Additional file 1: Supplementary Figure S1A, S1B). DLC1 is haploinsufficient in the mammary gland [16] and functions as a tumor suppressor through enhanced Rho signaling when deleted [17]. To test for other genomic changes with the potential to increase Rho signaling, we looked for chromosome losses that include genes with RhoGAP-like domains. Indeed, more than 40% of breast tumors in The Cancer Genome Atlas (TCGA) cohort show deletions that included *ARHGAP44* and *ABR* on 17p, *ARHGAP20* and *ARHGAP32* on 11q, *STARD13/DLC2* on 13q, as well as *PRR5/ARHGAP8*, *SH3BP1* and *BCR* on 22q (Additional file 1: Supplementary Figure S1A, S1B). Copy number gains and structural variants in RhoGEF genes were also evident, many of which have the potential to increase Rho signaling through increased GTP-loading [6]. For example, over 50% of TCGA breast tumors show copy number gains or amplifications involving *OBSCN, ARHGEF2* and *ARHGEF11* on 1q or *PREX2* on 8q (Additional file 1: Supplementary Figure S1C, S1D). Next, we looked for SNV or copy number changes in genes coding for Rho-family proteins. While SNVs were uncommon, copy number gains were seen. For example, *RHOC* gains were found in 4 (METABRIC) to 16 (TCGA) percent of cases (Fig. 1A and Additional file 1: Supplementary Figure S2A). In comparison to controls, a greater percentage of tumors with increased *RHOC* gene copies were ERα-negative (Fig. 1A) and, in the case of METABRIC cohort tumors, associated with a significant increase in *RHOC* gene expression (Fig. 1B). In the TCGA cohort, a trend towards increased expression was seen for tumors with copy number gains or amplifications which included the RHOC gene, although this did not reach significance (Additional file 1: Supplementary Figure S2B). Finally, more tumors with *RhoC* Gains/Amplifications were of the basal subtype in comparison to tumors without copy number gains for *RhoC* (Fig. 1C). Also, more were Histological Grade 3 (Fig. 1D).

### Generation of a *cre*-conditional *RhoC* transgenic line

It is well established that RhoGAPs can function as tumor suppressors, whereas RhoGEFs and activated Rho mutants can be oncogenes [2, 18]. The importance of increased Rho protein expression is less clear. Indeed, widespread expression of GEFs and GAPs suggests that RHO proteins are regulated mostly at the level of GTP-loading. Despite this, there are situations where Rho expression is limiting [10, 11]. To study elevated *RhoC* expression in vivo, we used gene targeting in embryonic stem cells to generate a Rosa26-based transgenic with *RhoC* linked through IRES sequences to eGFP downstream of a loxP-stop-loxP cassette (Additional file 1: Supplementary Figure S3) [19, 20].

### *RhoC* overexpression cooperates with *ErbB2/Neu* to induce mammary tumor formation

*RhoC* overexpression and *ErbB2/Neu* status are positively correlated in invasive carcinoma [21]. However, potential cooperation between *RhoC* overexpression and *ErbB2/Neu* has never been directly studied in vivo. To test for this, we crossed our *RhoC* transgenics to two different models for *ErbB2/Neu*^+^ breast cancer: *FloxNeoNeu*^*NT*^ (with an activated *Neu*^*NT*^ cDNA targeted to the mouse *ErbB2* locus but preceded by loxP-stop-loxP sequences) [22] and NIC (where a *Neu*^*NDL2-5*^-IRES-Cre transcript is regulated by the MMTV LTR) [23]. Previous work has shown that *Cre-*dependent *FloxNeoNeu*^*NT*^ mice develop mammary tumors at a mean age of 15 months [22], whereas MMTV-NIC mice develop tumors as early as 4 months [23]. *FloxNeoNeu*^*NT*^;MMTV*-Cre* mammary tumors, for the most part, do not metastasize [22]. For our experiments, we used MMTV-Cre^NLST^ to activate *Neu*^*NT*^ expression in *FloxNeoNeu*^*NT*^ mice [24]. This Cre transgenic line is mammary-specific but appears to express in fewer mammary epithelial cells or at a lower level in mammary epithelium than other MMTV-Cre delete strains, including MMTV-Cre^Line7^, which was used previously to activate Neu^NT^ expression in FloxNeoNeu^NT^ mice [22]. Indeed, only 4/30 *FloxNeoNeu*^*NT*^;MMTV*-Cre*^NLST^ mice even developed mammary tumors, and all of these occurred in very old animals (Additional file 1: Supplementary Figure S4A and S4B). Mammary tumors in these mice were predominantly squamous (Additional file 1: Supplementary Figure S4C).

**Fig. 2:**
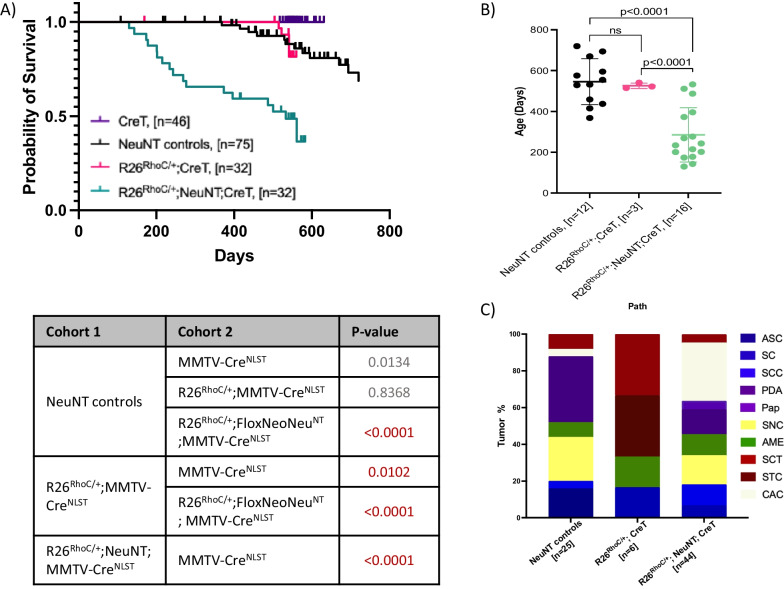
RhoC overexpression cooperates with endogenously driven activated Neu (FloxNeoNeu^NT^) to enhance tumor formation. **A** Kaplan Meier survival curve showing cooperation between FloxNeoNeu^NT^ and RhoC overexpression. Death due to mammary tumor end-point was compared between cohorts. Statistical analysis for KM survival curves was calculated using Log-rank (Mantel-Cox) test via GraphPad Prism (shown in the table below) and p-values of less than 0.05 are considered significant (red text). **B** Graph comparing the ages between cohorts at the end-point due to mammary gland tumors. **C** The column graph shows the mammary tumor histology. Tumor types are represented with different colors. Mammary tumor histotypes are divided as below. ASC, Adenosquamous carcinoma; SC, Squamous cyst; SCC, Squamous cell carcinoma; PDA, Poorly differentiated adenocarcinoma; Pap, Papillary adenocarcinoma; SNC, Solid nodular carcinoma; AME, Adenomyoepithelioma; CAC, Complex adenocarcinoma; STC, Scirrhous tubular carcinoma; SCT, Spindle cell tumor. FloxNeoNeu^NT^ controls (or Neu^NT^ controls) contain data from FloxNeoNeu^NT^;MMTV-Cre^NLST^ and *Gata4*^*loxP/*+^;FloxNeoNeu^NT^;MMTV-Cre^NLST^ cohorts (for separate analysis of these cohorts, see Additional file 1: Supplementary Figure S4). Note: CreT = Cre^NLST^

Many mammary tumors that form in *FloxNeoNeu*^*NT*^ model mice select for amplification of the *ErbB2/Neu*^*NT*^ locus [22]. In fact, it has been suggested that amplification of *ErbB2* is a mechanism to circumvent repression of the *ErbB2* promoter by Gata4 and other DNA-binding proteins [25, 26]. We tested for this by deleting one copy of *Gata4* in this model. While trending towards decreased latency, mammary tumor formation in *Gata4*^*loxP/*+^;*FloxNeoNeu*^*NT*^;MMTV*-Cre*^NLST^ mice was not significantly different than seen in *FloxNeoNeu*^*NT*^;MMTV*-Cre*^NLST^ controls (Additional file 1: Supplementary Figure S4A and S4B). Most tumors that formed in *Gata4*^*loxP/*+^;*FloxNeoNeu*^*NT*^;MMTV*-Cre*^NLST^ mice were either poorly differentiated adenocarcinomas or solid nodular carcinomas (a histology commonly associated with transformation by activated Neu [23, 27]) (Additional file 1: Supplementary Figure S4C). As tumor latency was not significantly affected by heterozygous deletion of *Gata4*, both cohorts (*FloxNeoNeu*^*NT*^;MMTV*-Cre*^NLST^ and *Gata4*^*loxP/*+^;*FloxNeoNeu*^*NT*^;MMTV*-Cre*^NLST^) were combined and used as controls for the effect of *RhoC* in a greater number of animals (see “NeuNT controls” below and in Fig. 2A).

Ectopic expression of *RhoC* dramatically reduced tumor-free survival (Fig. 2A). In addition, R26^*RhoC/*+^;*FloxNeoNeu*^*NT*^;MMTV*-Cre*^NLST^ mice developed mammary tumors much faster than controls: as early as 4.5 months, whereas the average age at which tumors formed in control mice was close to a year and a half (Fig. 2B). While not significant, a trend towards an increased number of R26^*RhoC/*+^;*FloxNeoNeu*^*NT*^;MMTV*-Cre*^NLST^ mice with metastasis was also seen (Additional file 1: Supplementary Figure S5A, S5B). On a NIC background, *RhoC* did not significantly alter mammary tumor-free survival curves, although *RhoC*-NIC model mice did die from mammary tumors at a significantly younger age than NIC controls (Additional file 1: Supplementary Figure S6A and S6B). This relatively subtle effect is likely related to the short latency for tumor formation in this model. Tumors in NIC model mice, with or without ectopic RhoC expression were almost exclusively solid nodular carcinomas (Additional file 1: Supplementary Figure S6C).

### Elevated wildtype RhoC enhances EMT signaling in *FloxNeoNeu*^*NT*^ model tumors

As noted above, high-level expression of activated *ErbB2/Neu* induces solid nodular carcinomas (SNC) in the mouse mammary gland [23, 27]. In contrast, activated *ErbB2/Neu* when expressed at a lower level in *FloxNeoNeu*^*NT*^:*MMTV-Cre*^*NLST*^ mice resulted in tumors with multiple different histologies [22]. Enhanced mammary tumor formation in R26^*RhoC/*+^;*FloxNeoNeu*^*NT*^;MMTV*-Cre*^NLST^ mice raises the possibility that RhoC-expression could alleviate a requirement for transgene amplification, at least not to the same extent as seen in our combined control cohort tumors. Therefore to assess amplification of the *ErbB2/Neu*^*NT*^ locus in R26^*RhoC/*+^;*FloxNeoNeu*^*NT*^;MMTV*-Cre*^NLST^ and controls, we used digital droplet PCR-based copy number analysis for *ErbB2/Neu*^*NT*^ and *Grb7* (the neighboring gene). Indeed, R26^*RhoC/*+^;*FloxNeoNeu*^*NT*^;MMTV*-Cre*^NLST^ tumors had a mean of 5.5 and 3.8 copies of *ErbB2/Neu*^*NT*^ and *Grb7*, respectively. In contrast, NeuNT controls showed an average of 618 and 676 copies. While these mean values appear very different, due to the wide variation seen for copy number changes at the *ErbB2/Neu*^*NT*^ locus in controls, these differences are not significant (Additional file 1: Supplementary Figure S7).

**Fig. 3:**
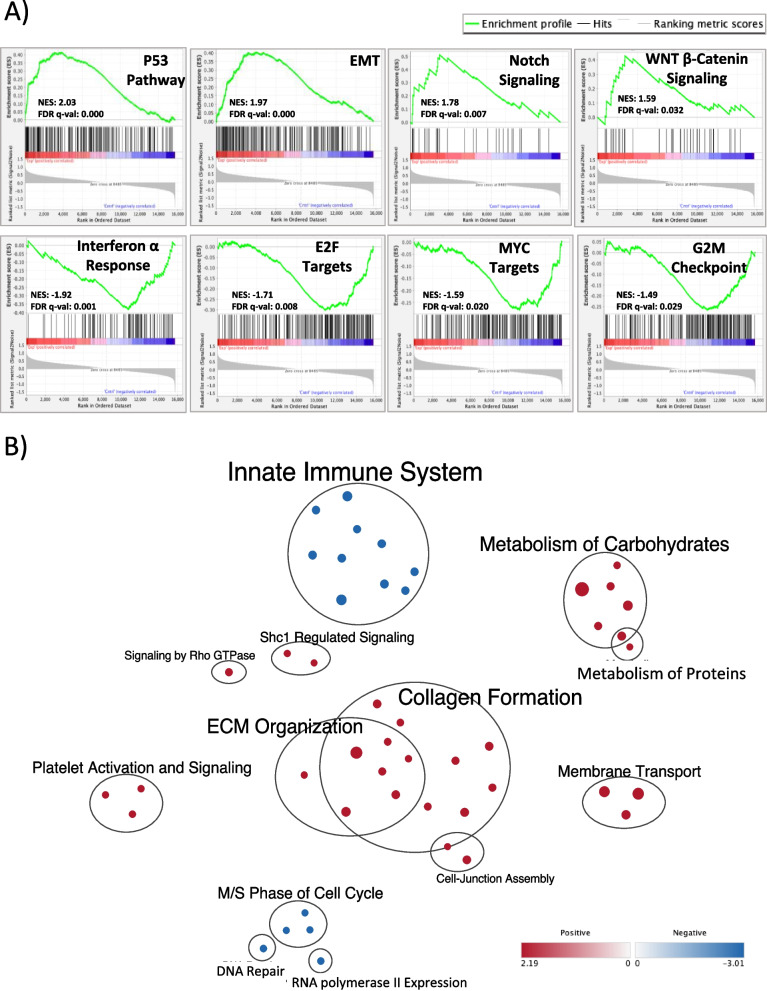
Gene Set Enrichment Analysis (GSEA) of RNA sequencing data from mammary tumor samples. **A** Enrichment plots profiling GSEA analysis based on mouse hallmark gene sets using differential gene expression data from R26^RhoC/+^; FloxNeoNeuNT; MMTV-Cre^NLST^ (experimental) mammary tumors compared to NeuNT controls. Gene expression associated with activation of p53, EMT, Notch and WNT/β-Catenin pathways was increased in the experimental group, while Interferon α response, E2F targets, Myc targets and G2M checkpoint pathways were increased in the control group. Only the top 4 up-/down-regulated pathways were shown and the rest can be found in Additional file 1: Supplementary Table 3. **B** Enrichment map visualization of the enriched pathways in mammary tumors from experimental and control mice. Nodes in the network represent pathways (Reactome, Biocarta, Wiki Pathways) and similar pathways with many common genes are connected. Node size is proportional to the number of genes in each node and colors indicate whether the member genes of a set are up (red) or down (blue) regulated in the experimental group compared to controls

Most mammary tumors in *FloxNeoNeu*^*NT*^ control mice were poorly differentiated adenocarcinomas, solid nodular carcinomas, or tumors with squamous differentiation (Fig. 2C). A similar mix was seen in R26^*RhoC/*+^;*FloxNeoNeu*^*NT*^;MMTV*-Cre*^NLST^ mice, although many tumors in this cohort showed a heterogeneous or complex histological pattern (Fig. 2C). Next, to identify transcriptional changes linked to RhoC-mediated accelerated mammary tumor formation, we performed bulk RNA-seq analysis on tumors from R26^*RhoC/*+^;*FloxNeoNeu*^*NT*^;MMTV*-Cre*^NLST^ and control cohorts (Additional file 1: Supplementary Table S1). Differential gene expression analysis was then performed using the DESeq2 tool within R. Tumors from the same cohorts clustered together by principal component analysis (PCA). Next, we performed pathway enrichment analysis using GSEA (Additional file 1: Supplementary Tables S2 and S3) and gProfiler (Additional file 1: Supplementary Tables S4). GSEA does not require a threshold to categorize differentially and non-differentially expressed genes. Therefore, the complete gene list identified from DESeq2 analysis was used. EMT, p53, Notch and WNT/β-catenin pathway signatures were increased in RhoC cohort (R) tumors, while Interferon α/Immune responses, E2F targets, Myc targets and G2M checkpoint pathways were decreased (Fig. 3A as well as Additional file 1: Supplementary Tables S2 and S3). EMT signature changes included significantly altered expression of *Dst*, *Msx1*, *P3h1*, *Notch2*, *Magee1*, *Tgfb1*, *Serpinh1*, *Tnc*, *Fbln2* and *Bmp1* (Fig. 3B and Additional file 1: Supplementary Table S2). Consistent with the trend towards lower-level *ErbB2*/*Grb7* copy number gains/amplification in R26^*RhoC/*+^;*FloxNeoNeu*^*NT*^;MMTV*-Cre*^NLST^ tumors, many of the genes near *ErbB2* were expressed at a lower level in RhoC tumors as compared to controls, while *ErbB2*/*Neu* mRNA levels were similar to what was seen in control tumors (Additional file 1: Supplementary Figure S8).

Finally, Rho mutant oncogenes have been identified in some human tumors. To test for the selection of activating mutations within the RhoC transgene, we used PCR-sequencing. No such mutations could be identified in 14 tumors from R26^*RhoC/*+^;*FloxNeoNeu*^*NT*^;MMTV*-Cre*^NLST^ mice, indicating that wildtype *RhoC* was responsible for accelerating mammary tumor formation (Additional file 1: Supplementary Table S5).

### *RhoC* overexpression cooperates with *Pik3ca*^*H1047R*^

**Fig. 4:**
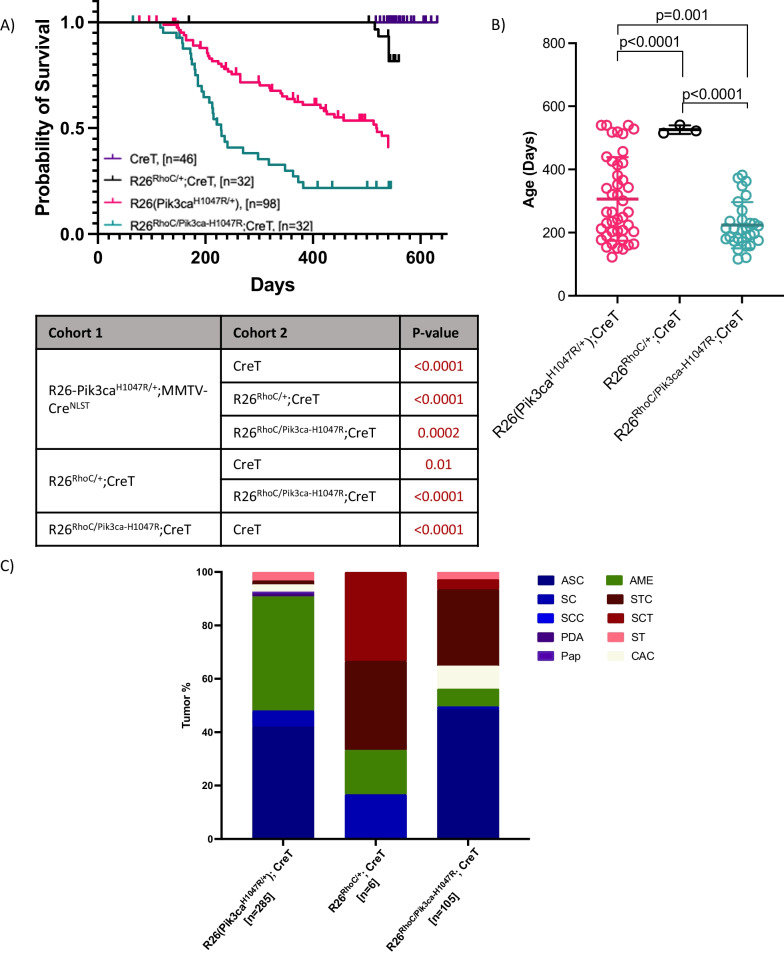
*RhoC* overexpression cooperates with activated *Pik3ca* (H1047R mutant) to enhance tumor formation. **A** Kaplan Meier survival curve showing cooperation between *Pik3ca*^*H1047R*^ and *RhoC* overexpression. Death due to mammary tumor progression was compared between cohorts. Statistical analysis for KM survival curves were calculated using Log-rank (Mantel-Cox) test via GrapPad Prism (shown in the table below) and *p*-values of less than 0.05 are considered significant. **B** Graph comparing the ages between cohorts when mice died due to mammary gland tumors. **C** The column graph shows mammary tumor histology for each cohort. Tumor types are represented with different colors. Mammary tumor histotypes are divided as below. ASC, Adenosquamous carcinoma; SC, Squamous cyst; SCC, Squamous cell carcinoma; PDA, Poorly differentiated adenocarcinoma; Pap, Papillary adenocarcinoma; AME, Adenomyoepithelioma; STC, Scirrhous tubular carcinoma; SCT, Spindle cell tumor; ST, Scirrhous tumor; CAC, Complex adenocarcinoma. Note: CreT = CreNLST

*PIK3CA* mutations are frequently seen in breast cancer. Therefore to test for the effect of RhoC overexpression on mammary tumor induction by a different oncogenic driver, we also crossed R26^*RhoC*^ mice to our model for *PIK3CA*-mutant breast cancer (R26-*Pik3ca*^*H1047R*^*;*MMTV*-Cre*^NLST^) [28]. 55% of R26-*Pik3ca*^H1047R^/*RhoC*;MMTV*-Cre*^NLST^ mice developed mammary tumors, a similar proportion to that seen in R26-*Pik3ca*^*H1047R*^;MMTV*-Cre*^NLST^ controls (45%). However, *Pik3ca*^*H1047R*^/*RhoC* mice reached endpoint with mammary tumors, on average, 100 days earlier than seen in *Pik3ca*^*H1047R*^ mice (Fig. 4A and B). Most mutant *Pik3ca* tumors were adenosquamous carcinomas (42%), Adenomyoepitheliomas (AMEs) (43%), or Squamous Cysts (SCs)(6%) (Fig. 4C). In contrast, R26-*Pik3ca*^*H1047R*^/*RhoC*;MMTV*-Cre*^NLST^ mice developed more spindle-family tumors (Fig. 4C). A coincidental reduction in the percentage of AMEs was evident (Fig. 4C). This result is also consistent with induction of EMT signature gene expression as seen in *FloxNeoNeu*^*NT*^ model tumors discussed above.

### *RhoC* overexpression does not enhance ErbB2 gene expression or PI3K/Akt signaling in T47D cells

One possible explanation for *RhoC* overexpression cooperating with *ErbB2*/*Neu* and *Pik3ca* oncogenes in transformation of mammary epithelium could involve RhoC-mediated enhancement of *ErbB2* expression and/or PI3K to Akt signaling. To test for this, we assessed the effect of RhoC on both parameters in transiently transfected T47D breast cancer cells. This cell line was chosen since it expresses *ErbB2* [29] and has an H1047R mutation in *PIK3CA* [30]. Despite overexpression of ErbB2/Neu^NT^ in R26^*RhoC/*+^; *FloxNeoNeu*^*NT*^;MMTV*-Cre*^NLST^ tumors without apparent selection for high-level amplification of *ErbB2*/*Neu*^*NT*^ (see Additional file 1: Supplementary Figure S9)), overexpression of RhoC did not enhance ERBB2 protein accumulation in transfected cells (Additional file 1: Supplementary Figure S9). Similarly, based on Threonine 308 or Serine 473 phosphorylation of Akt proteins (Additional file 1: Supplementary Figure S9), overexpression of RhoC also did not significantly enhance PI3K to Akt signaling. Thus, while RhoC overexpression cooperates with both oncogenic proteins/pathways, this effect is not easily modeled in vitro and may well relate to non-cell-autonomous effects of RhoC in the tumor microenvironment.

## Summary

*RhoC* overexpression in BC was first identified in a clinical subtype known as Inflammatory Breast Cancer [31]. In vitro, increased RhoC protein levels lead to transformation and invasion of HME and MCF-10A cells [11, 32–34]. Despite this, the role of *RhoC* overexpression in transformation of mammary epithelial cells in vivo has not been addressed. Here, we report on generation and characterization of a *Cre-*conditional *RhoC*-overexpression mouse. To test for the transforming effect of overexpression on different oncogenic backgrounds, R26-*RhoC* mice were crossed to *ErbB2/Neu*^*NT/NDL2-5*^ and *Pik3ca*^*H1047R*^ models of BC. *ERBB2/Neu* gain or amplification occurs in approximately 25–30% of human breast tumors. *PIK3CA* is activated through mutation in ~ 35% of cases, most of which do not show amplification of *ErbB2*. Thus, collectively, *ERBB2/Neu*^+^ and *PIK3CA*^mutant^ breast tumors represent the majority of cases. We therefore chose to study *RhoC* overexpression in models for both alterations. Indeed, RhoC overexpression dramatically increased mammary tumor formation induced by *Neu*^*NT*^ and *Pik3ca*^*H1047R*^. RhoC overexpression did not affect ERBB2 protein accumulation in transfected breast cancer cells in vitro (Additional file 1: Supplementary Figure S8). In addition, RhoC overexpression failed to enhance PI3K to Akt signaling in vitro (Additional file 1: Supplementary Figure S8). These data suggest that *RhoC* may cooperate with *ErbB2/Neu* and *Pik3ca* oncogenic signaling through a more indirect, even non-cell-autonomous, mechanism that is not easily modeled in vitro. Perhaps this mechanism may relate to the ability of RhoC to enhance motility or to a change in the tumor microenvironment associated with RhoC-mediated EMT in tumor cells. Indeed, *RhoC-ErbB2/Neu*^*NT*^ mammary tumors showed elevated EMT-associated gene expression, as well as elevated expression of p53, Notch- and Wnt-pathway genes. In *RhoC-Pik3ca*^*H1047R*^ mammary tumors, a shift in tumor histology was noted (in comparison to tumors that formed in control *Pik3ca*^*H1047R*^ model mice). This shift involved the development of spindle/EMT-like tumors at the expense of more benign Adenomyoepitheliomas. Thus, in cooperation with both oncogenes, RhoC enhanced epithelial to mesenchymal transition-associated properties. These data highlight the oncogenic effect of increased Rho expression in breast tumor formation, thereby revealing a potential benefit of targeting Rho protein expression in the clinic.

### Supplementary Information

**Additional file1** 

## Data Availability

RNA-Seq data are available at GEO, with the following accession number: GSE249601 (https://www.ncbi.nlm.nih.gov/geo/query/acc.cgi?acc=GSE249601).
