# Supplementary material for: Elevated expression of wildtype RhoC promotes ErbB2- and Pik3ca-induced mammary tumor formation
Source: Breast Cancer Res. 2024 May 28;26:86. doi: 10.1186/s13058-024-01842-5 (PMC11134842; doi:10.1186/s13058-024-01842-5)
Supplement: Supplementary file 1 — Additional file1 [file 13058_2024_1842_MOESM1_ESM.pptx]

## Slide 1
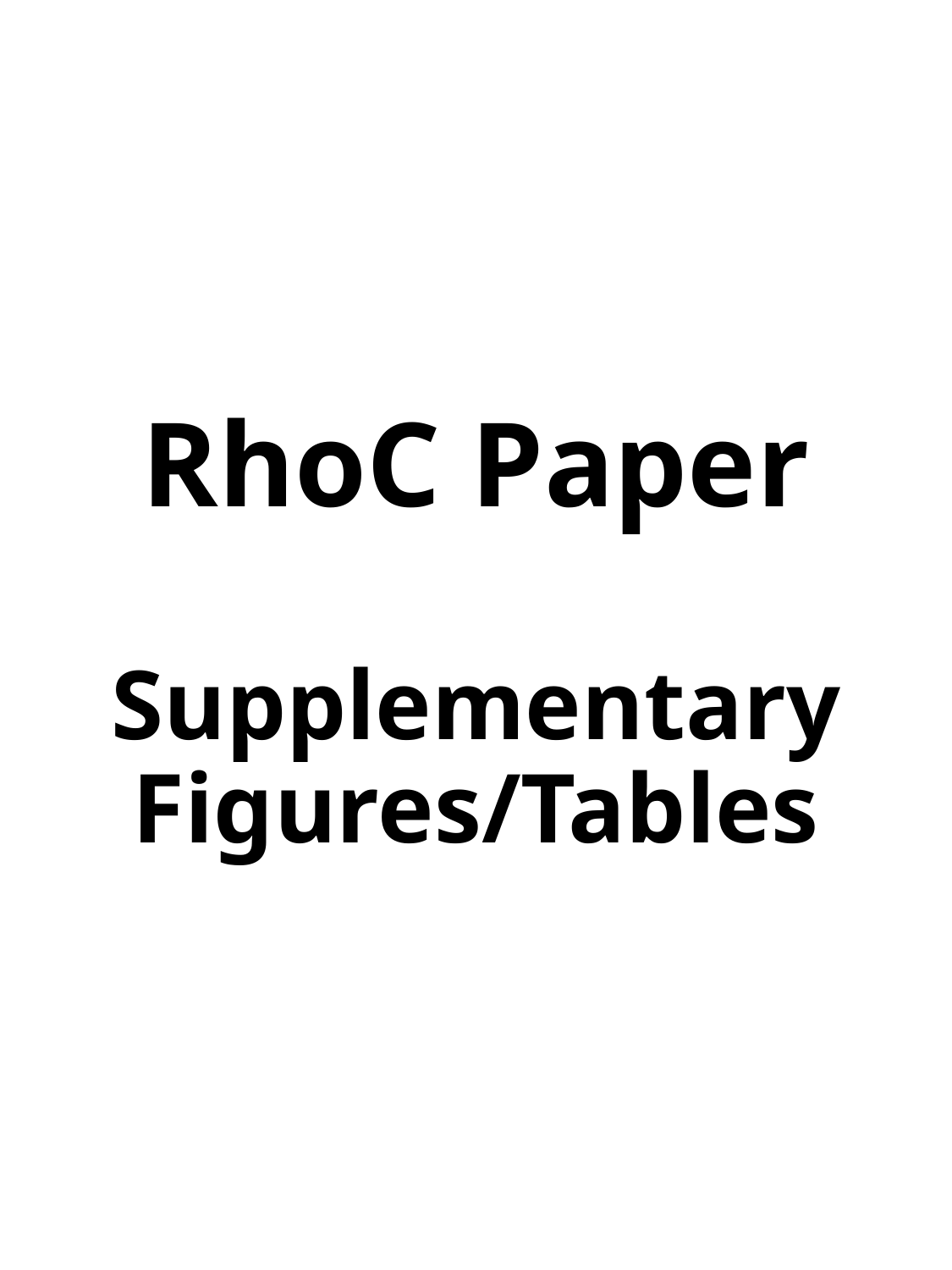

RhoC Paper
Supplementary Figures/Tables

## Slide 2
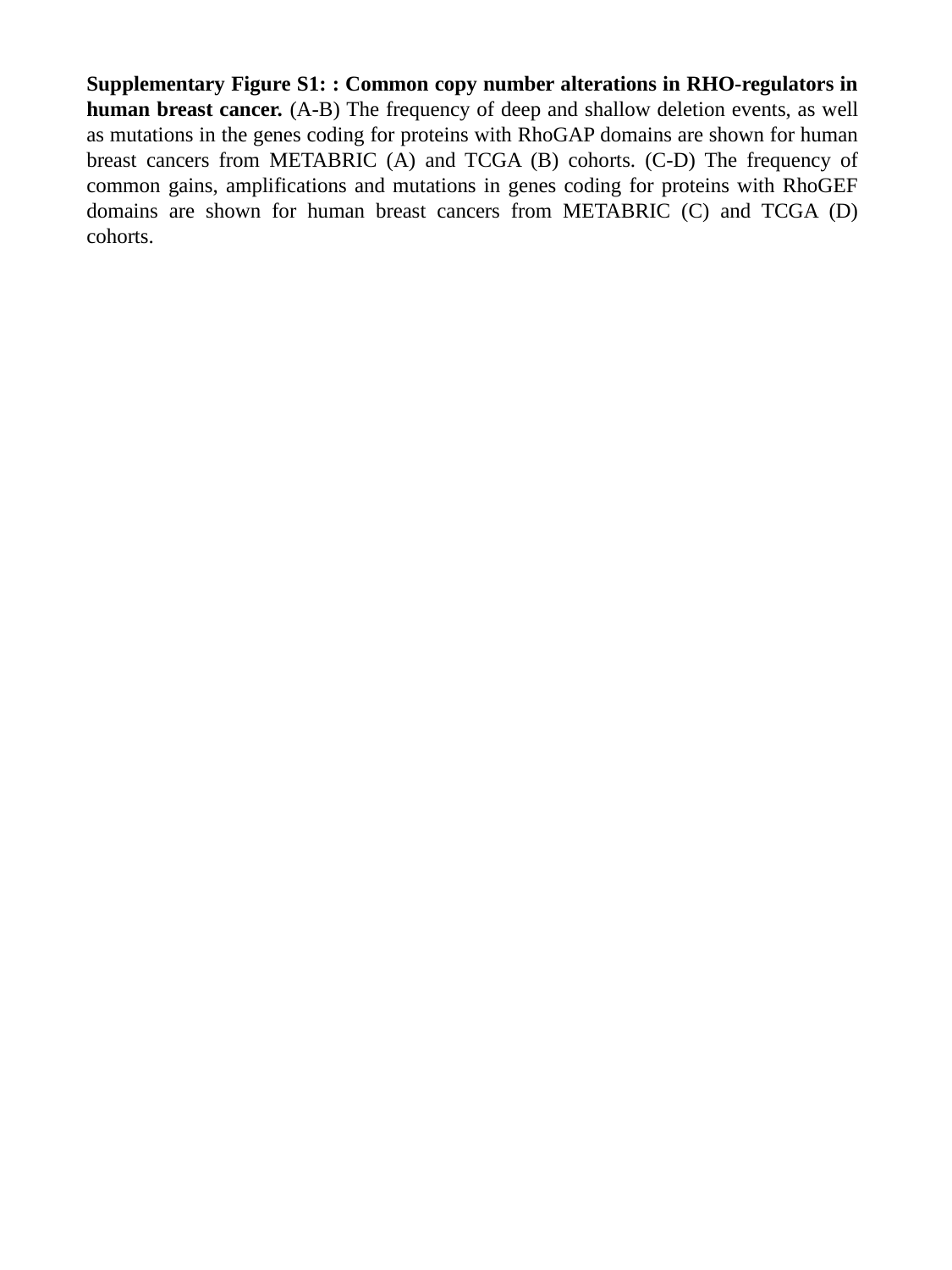

Supplementary Figure S1: : Common copy number alterations in RHO-regulators in human breast cancer. (A-B) The frequency of deep and shallow deletion events, as well as mutations in the genes coding for proteins with RhoGAP domains are shown for human breast cancers from METABRIC (A) and TCGA (B) cohorts. (C-D) The frequency of common gains, amplifications and mutations in genes coding for proteins with RhoGEF domains are shown for human breast cancers from METABRIC (C) and TCGA (D) cohorts.

## Slide 3
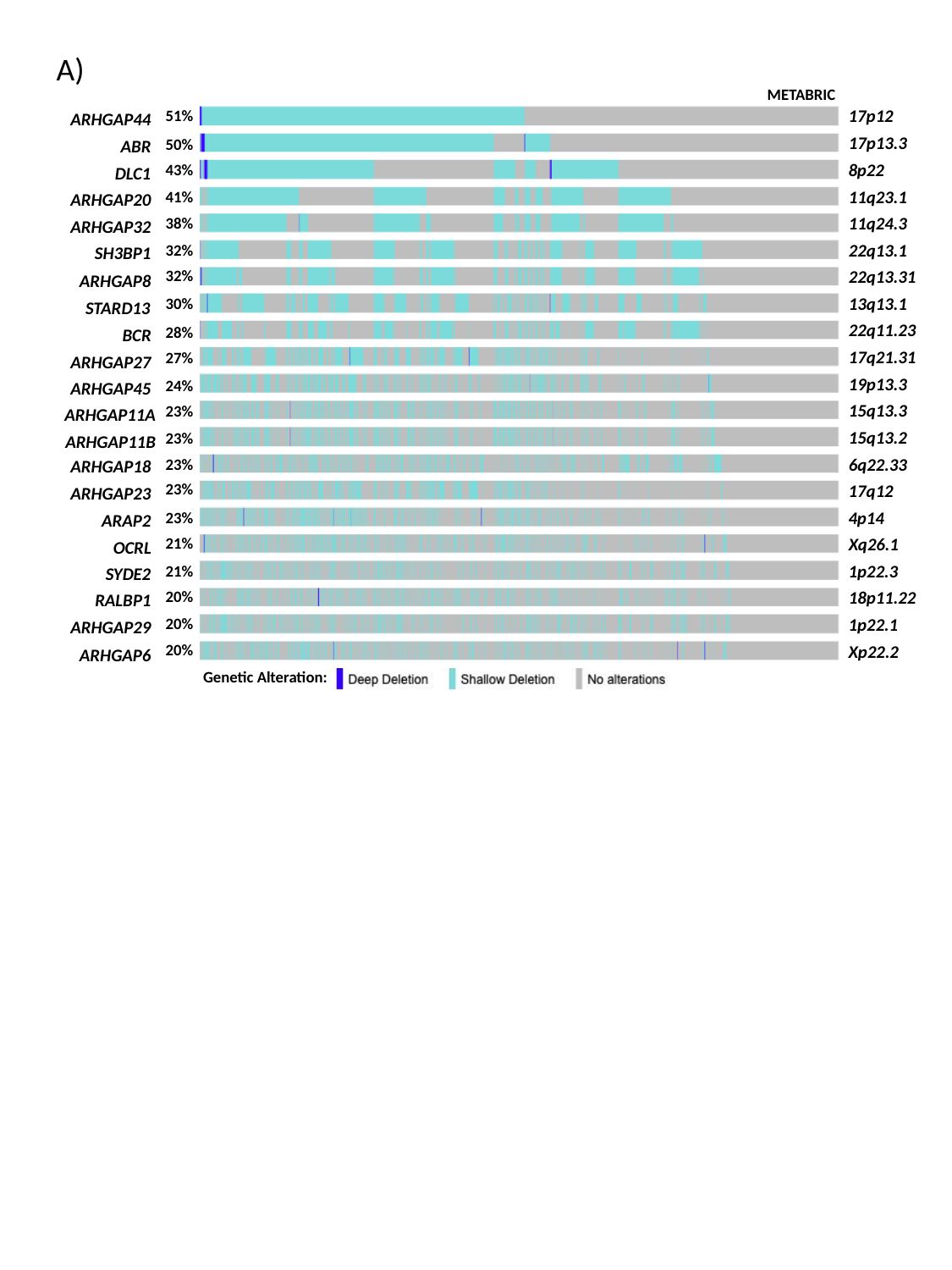

A)
METABRIC
17p12
ARHGAP44
51%
17p13.3
ABR
50%
8p22
DLC1
43%
11q23.1
ARHGAP20
41%
11q24.3
ARHGAP32
38%
22q13.1
SH3BP1
32%
22q13.31
ARHGAP8
32%
13q13.1
STARD13
30%
22q11.23
BCR
28%
17q21.31
ARHGAP27
27%
19p13.3
ARHGAP45
24%
15q13.3
ARHGAP11A
23%
15q13.2
ARHGAP11B
23%
6q22.33
ARHGAP18
23%
17q12
ARHGAP23
23%
4p14
ARAP2
23%
Xq26.1
OCRL
21%
1p22.3
SYDE2
21%
18p11.22
RALBP1
20%
1p22.1
ARHGAP29
20%
Xp22.2
ARHGAP6
20%
Genetic Alteration:

## Slide 4
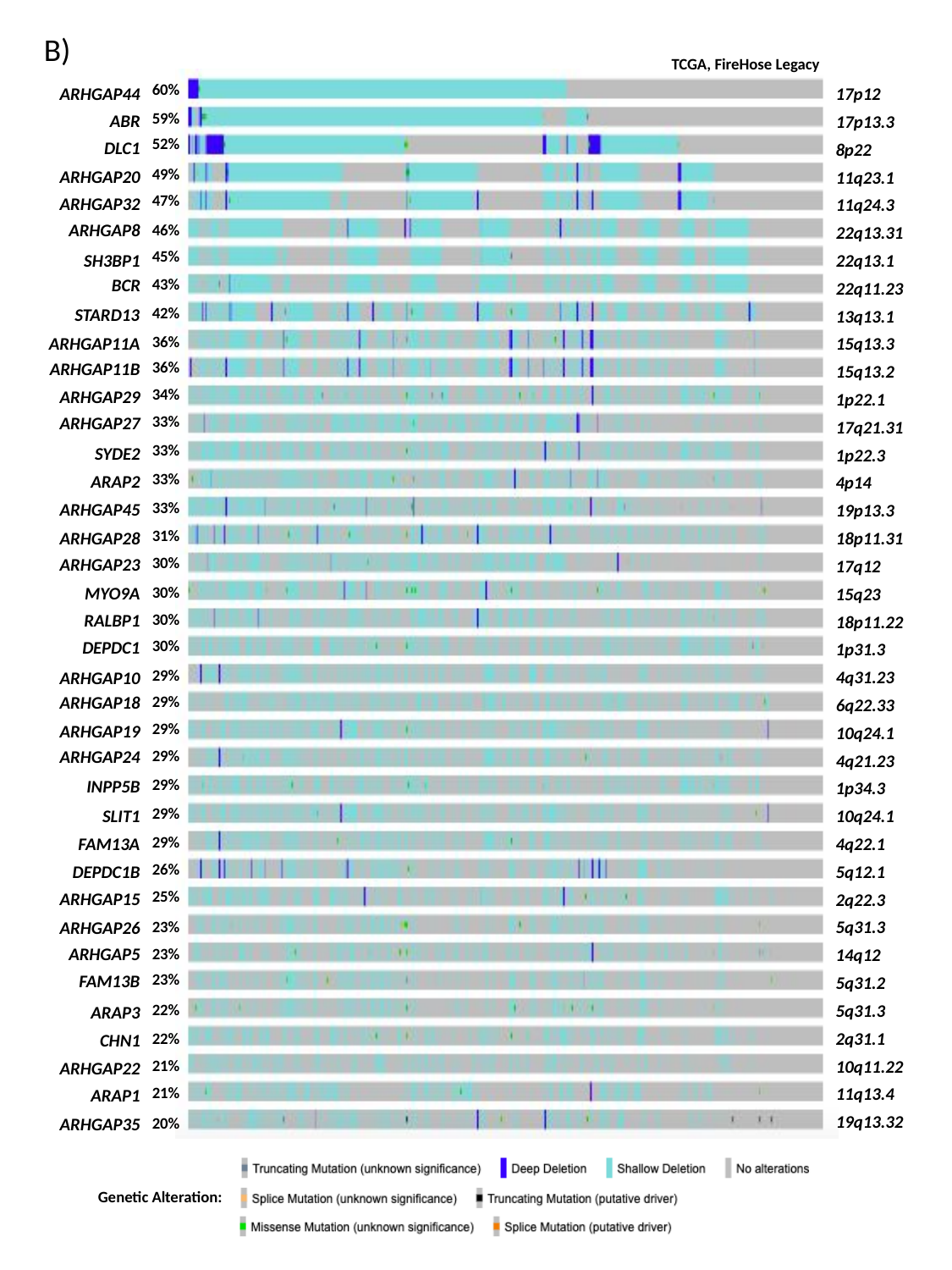

B)
TCGA, FireHose Legacy
ARHGAP44
17p12
60%
ABR
17p13.3
59%
DLC1
8p22
52%
ARHGAP20
11q23.1
49%
ARHGAP32
11q24.3
47%
ARHGAP8
22q13.31
46%
SH3BP1
22q13.1
45%
BCR
22q11.23
43%
STARD13
13q13.1
42%
ARHGAP11A
15q13.3
36%
ARHGAP11B
15q13.2
36%
ARHGAP29
1p22.1
34%
ARHGAP27
17q21.31
33%
SYDE2
1p22.3
33%
ARAP2
4p14
33%
ARHGAP45
19p13.3
33%
ARHGAP28
18p11.31
31%
ARHGAP23
17q12
30%
MYO9A
15q23
30%
RALBP1
18p11.22
30%
DEPDC1
1p31.3
30%
4q31.23
ARHGAP10
29%
ARHGAP18
6q22.33
29%
ARHGAP19
10q24.1
29%
ARHGAP24
4q21.23
29%
INPP5B
1p34.3
29%
SLIT1
10q24.1
29%
4q22.1
FAM13A
29%
5q12.1
DEPDC1B
26%
ARHGAP15
2q22.3
25%
5q31.3
ARHGAP26
23%
ARHGAP5
14q12
23%
FAM13B
5q31.2
23%
5q31.3
ARAP3
22%
2q31.1
CHN1
22%
10q11.22
ARHGAP22
21%
11q13.4
ARAP1
21%
19q13.32
ARHGAP35
20%
Genetic Alteration:

## Slide 5
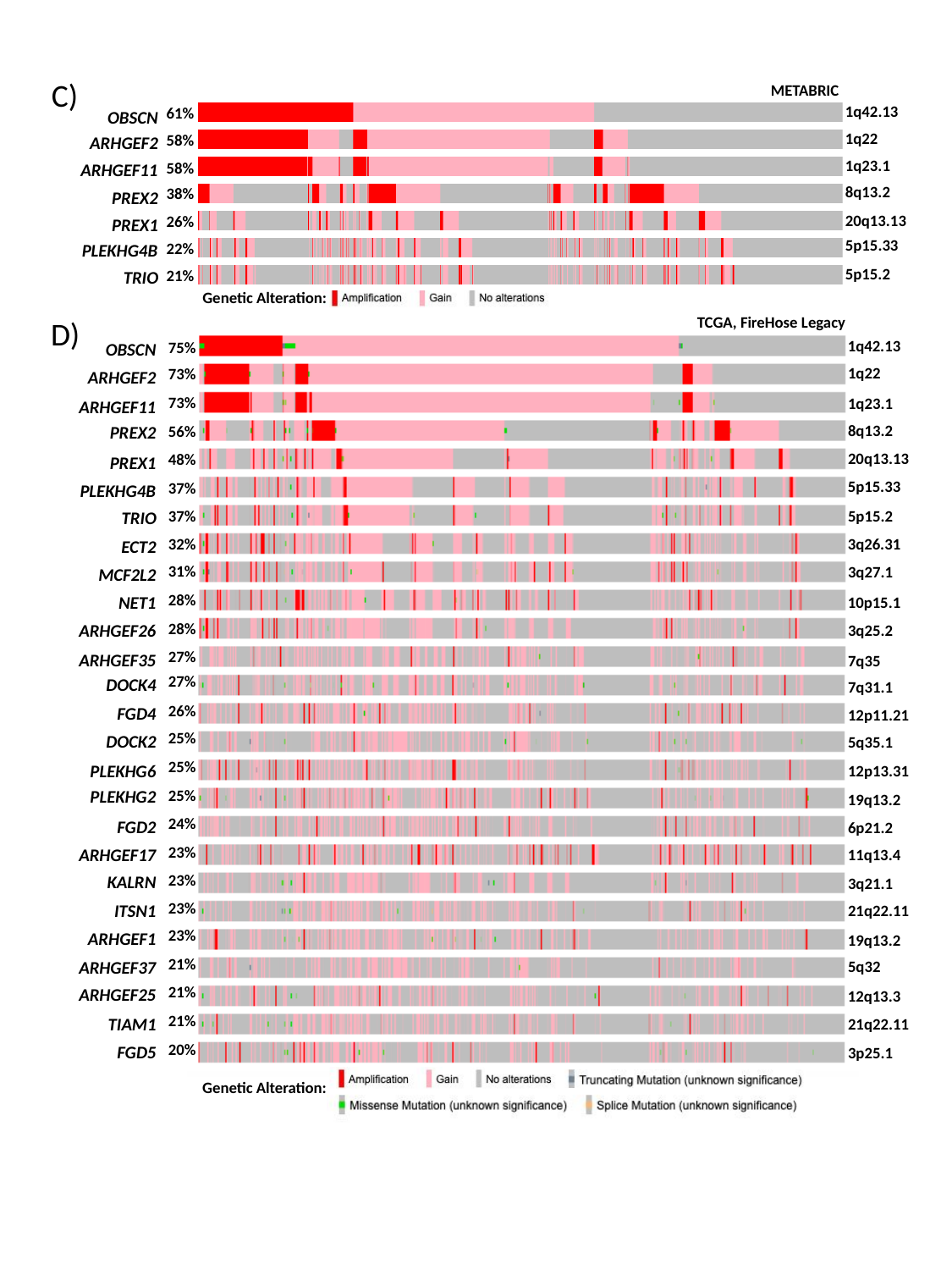

C)
METABRIC
OBSCN
1q42.13
61%
ARHGEF2
1q22
58%
ARHGEF11
1q23.1
58%
PREX2
8q13.2
38%
PREX1
20q13.13
26%
PLEKHG4B
5p15.33
22%
TRIO
5p15.2
21%
Genetic Alteration:
TCGA, FireHose Legacy
D)
OBSCN
1q42.13
75%
ARHGEF2
1q22
73%
ARHGEF11
73%
1q23.1
PREX2
8q13.2
56%
PREX1
20q13.13
48%
PLEKHG4B
5p15.33
37%
TRIO
37%
5p15.2
ECT2
3q26.31
32%
MCF2L2
31%
3q27.1
NET1
28%
10p15.1
ARHGEF26
28%
3q25.2
ARHGEF35
27%
7q35
DOCK4
27%
7q31.1
FGD4
26%
12p11.21
DOCK2
25%
5q35.1
PLEKHG6
25%
12p13.31
PLEKHG2
25%
19q13.2
FGD2
24%
6p21.2
ARHGEF17
23%
11q13.4
KALRN
23%
3q21.1
ITSN1
23%
21q22.11
ARHGEF1
23%
19q13.2
ARHGEF37
21%
5q32
ARHGEF25
21%
12q13.3
TIAM1
21%
21q22.11
FGD5
20%
3p25.1
Genetic Alteration:

## Slide 6
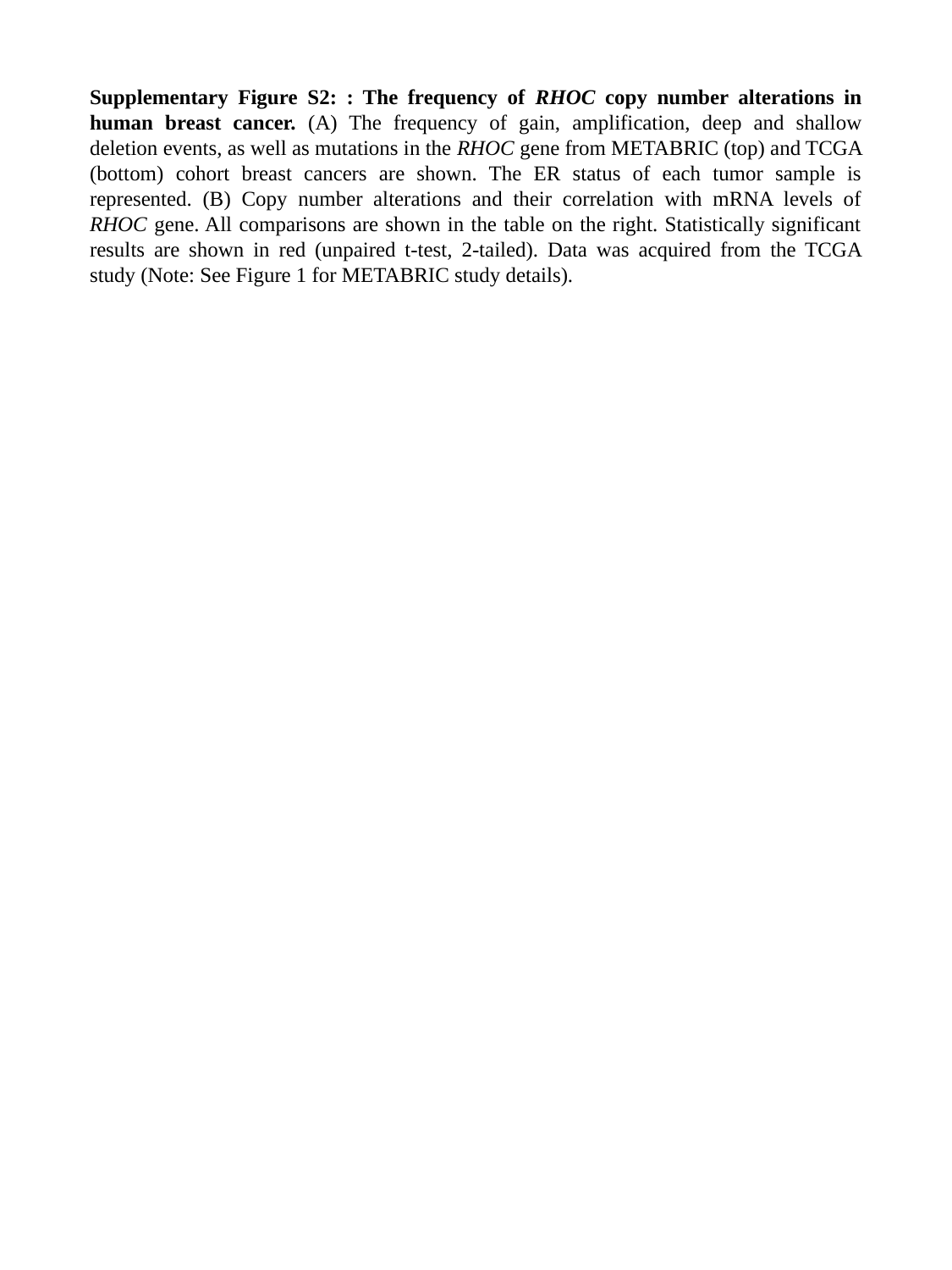

Supplementary Figure S2: : The frequency of RHOC copy number alterations in human breast cancer. (A) The frequency of gain, amplification, deep and shallow deletion events, as well as mutations in the RHOC gene from METABRIC (top) and TCGA (bottom) cohort breast cancers are shown. The ER status of each tumor sample is represented. (B) Copy number alterations and their correlation with mRNA levels of RHOC gene. All comparisons are shown in the table on the right. Statistically significant results are shown in red (unpaired t-test, 2-tailed). Data was acquired from the TCGA study (Note: See Figure 1 for METABRIC study details).

## Slide 7
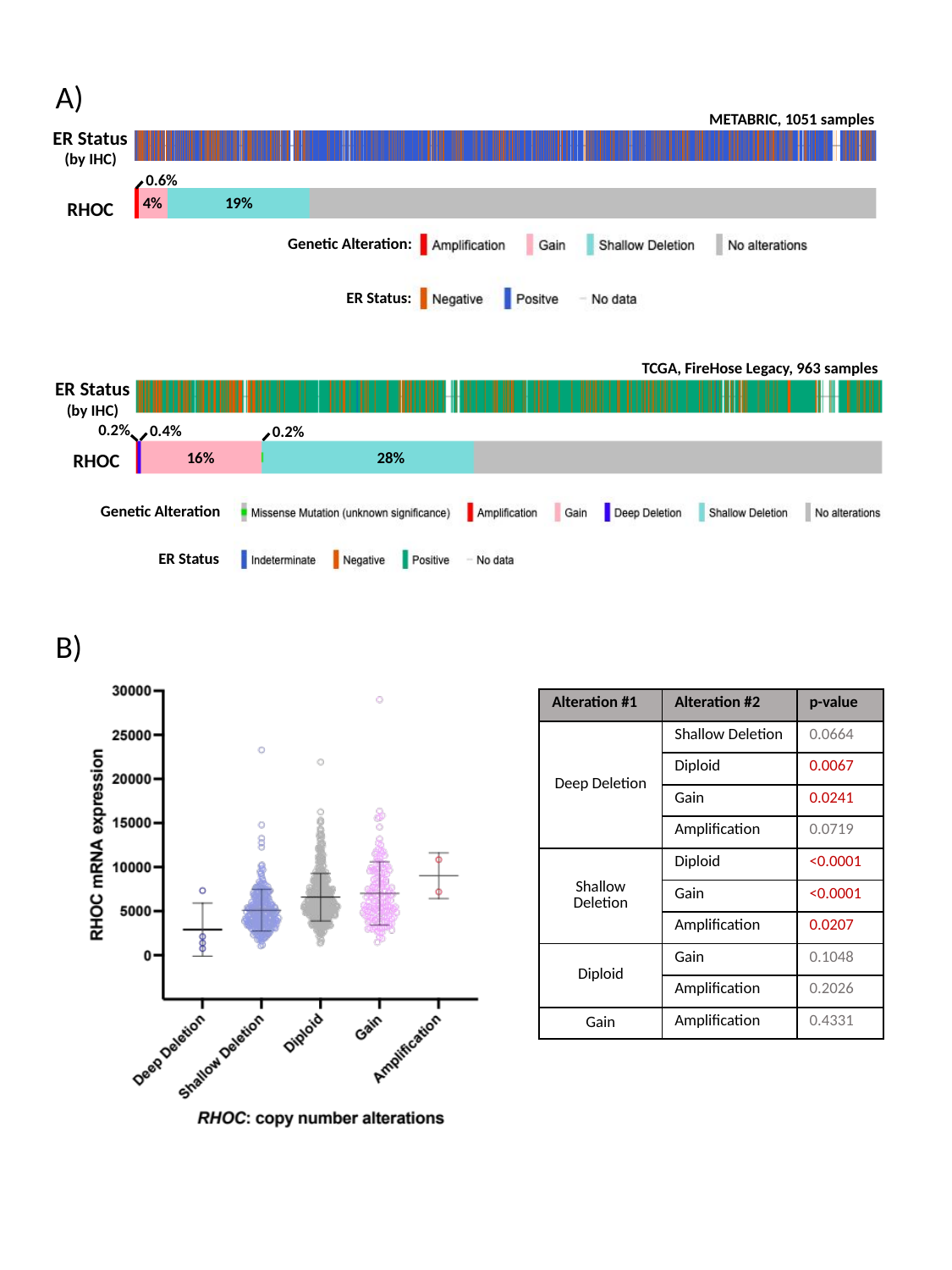

A)
METABRIC, 1051 samples
ER Status
(by IHC)
0.6%
RHOC
4%
19%
Genetic Alteration:
ER Status:
TCGA, FireHose Legacy, 963 samples
ER Status
(by IHC)
0.2%
0.4%
0.2%
RHOC
16%
28%
Genetic Alteration
ER Status
B)
| Alteration #1 | Alteration #2 | p-value |
| --- | --- | --- |
| Deep Deletion | Shallow Deletion | 0.0664 |
| | Diploid | 0.0067 |
| | Gain | 0.0241 |
| | Amplification | 0.0719 |
| Shallow Deletion | Diploid | <0.0001 |
| | Gain | <0.0001 |
| | Amplification | 0.0207 |
| Diploid | Gain | 0.1048 |
| | Amplification | 0.2026 |
| Gain | Amplification | 0.4331 |

## Slide 8
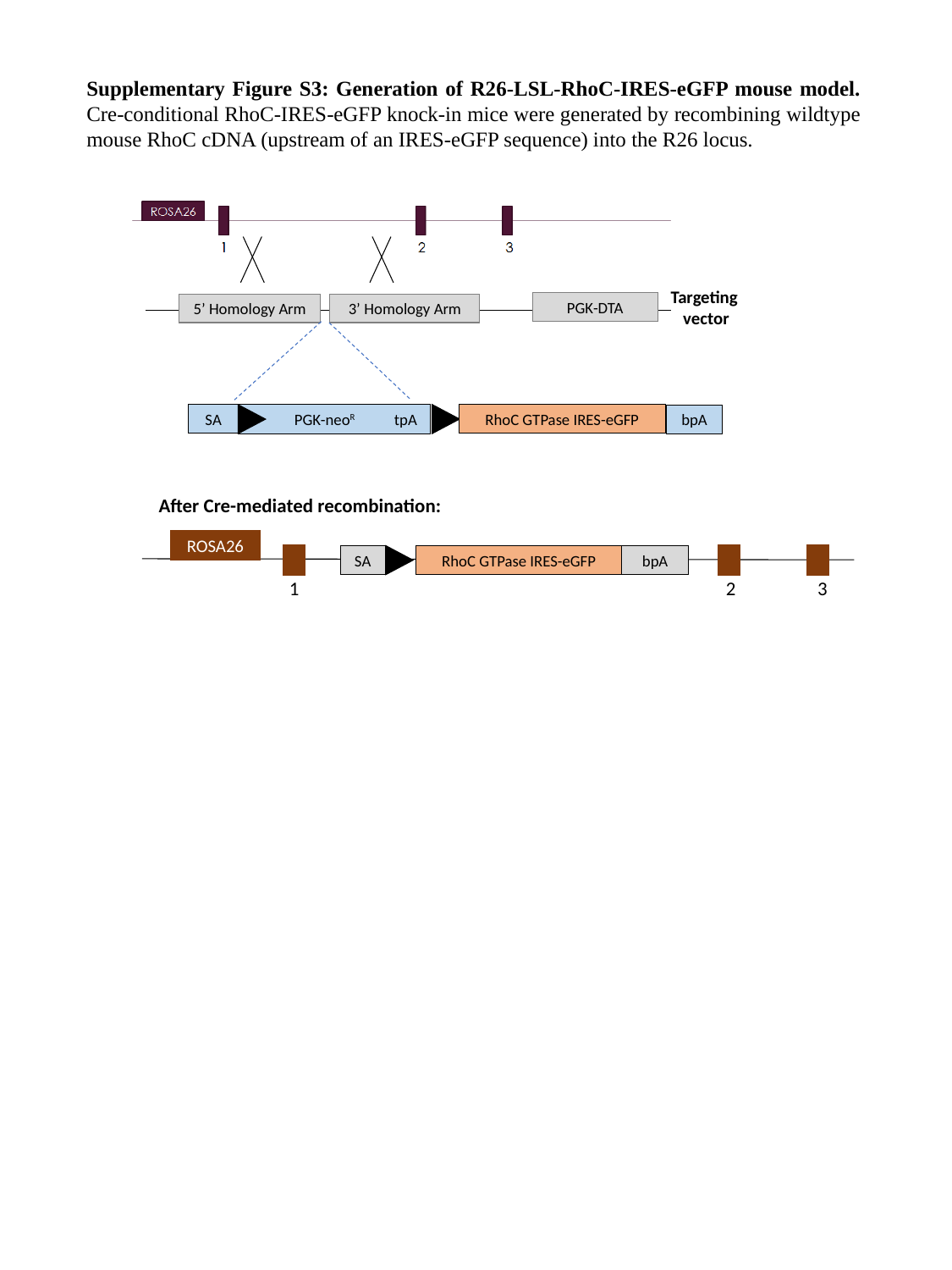

Supplementary Figure S3: Generation of R26-LSL-RhoC-IRES-eGFP mouse model. Cre-conditional RhoC-IRES-eGFP knock-in mice were generated by recombining wildtype mouse RhoC cDNA (upstream of an IRES-eGFP sequence) into the R26 locus.
Targeting
vector
PGK-DTA
5’ Homology Arm
3’ Homology Arm
SA
RhoC GTPase IRES-eGFP
 PGK-neoR tpA
bpA
After Cre-mediated recombination:
ROSA26
SA
RhoC GTPase IRES-eGFP
bpA
1
2
3

## Slide 9
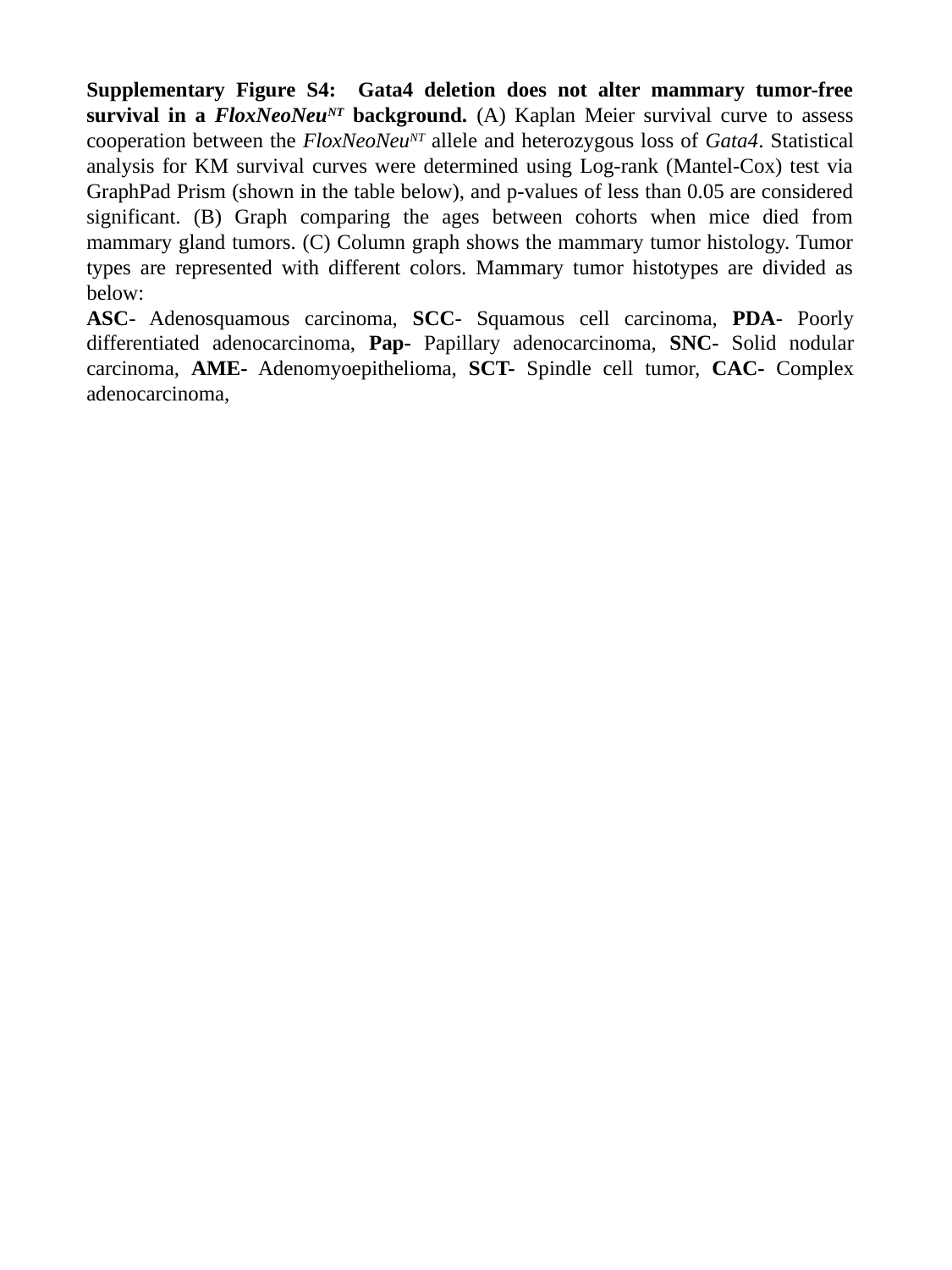

Supplementary Figure S4: Gata4 deletion does not alter mammary tumor-free survival in a FloxNeoNeuNT background. (A) Kaplan Meier survival curve to assess cooperation between the FloxNeoNeuNT allele and heterozygous loss of Gata4. Statistical analysis for KM survival curves were determined using Log-rank (Mantel-Cox) test via GraphPad Prism (shown in the table below), and p-values of less than 0.05 are considered significant. (B) Graph comparing the ages between cohorts when mice died from mammary gland tumors. (C) Column graph shows the mammary tumor histology. Tumor types are represented with different colors. Mammary tumor histotypes are divided as below:
ASC- Adenosquamous carcinoma, SCC- Squamous cell carcinoma, PDA- Poorly differentiated adenocarcinoma, Pap- Papillary adenocarcinoma, SNC- Solid nodular carcinoma, AME- Adenomyoepithelioma, SCT- Spindle cell tumor, CAC- Complex adenocarcinoma,

## Slide 10
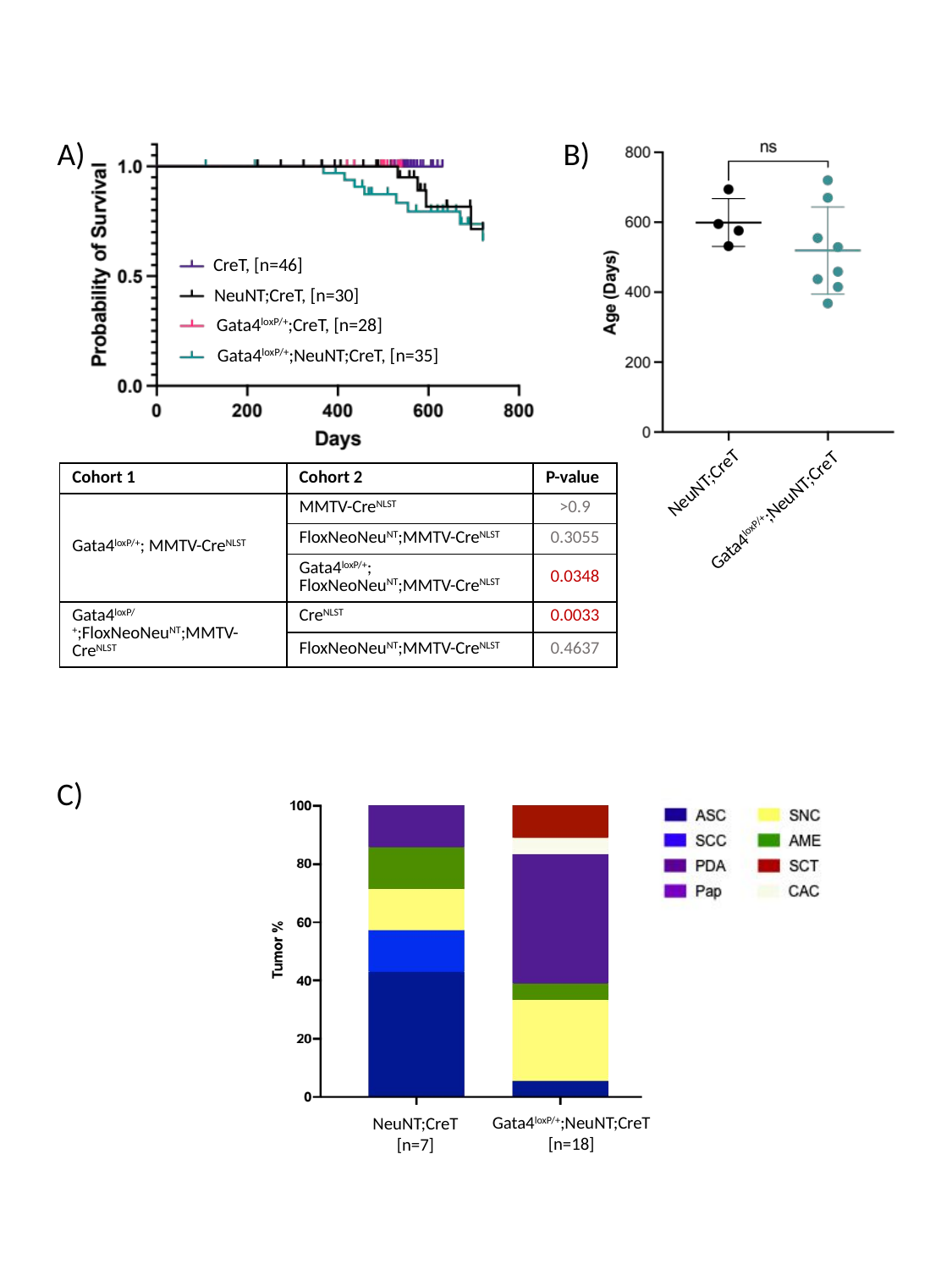

A)
B)
NeuNT;CreT
Gata4loxP/+;NeuNT;CreT
CreT, [n=46]
NeuNT;CreT, [n=30]
Gata4loxP/+;CreT, [n=28]
Gata4loxP/+;NeuNT;CreT, [n=35]
| Cohort 1 | Cohort 2 | P-value |
| --- | --- | --- |
| Gata4loxP/+; MMTV-CreNLST | MMTV-CreNLST | >0.9 |
| | FloxNeoNeuNT;MMTV-CreNLST | 0.3055 |
| | Gata4loxP/+; FloxNeoNeuNT;MMTV-CreNLST | 0.0348 |
| Gata4loxP/+;FloxNeoNeuNT;MMTV-CreNLST | CreNLST | 0.0033 |
| | FloxNeoNeuNT;MMTV-CreNLST | 0.4637 |
C)
Gata4loxP/+;NeuNT;CreT
[n=18]
NeuNT;CreT
[n=7]

## Slide 11
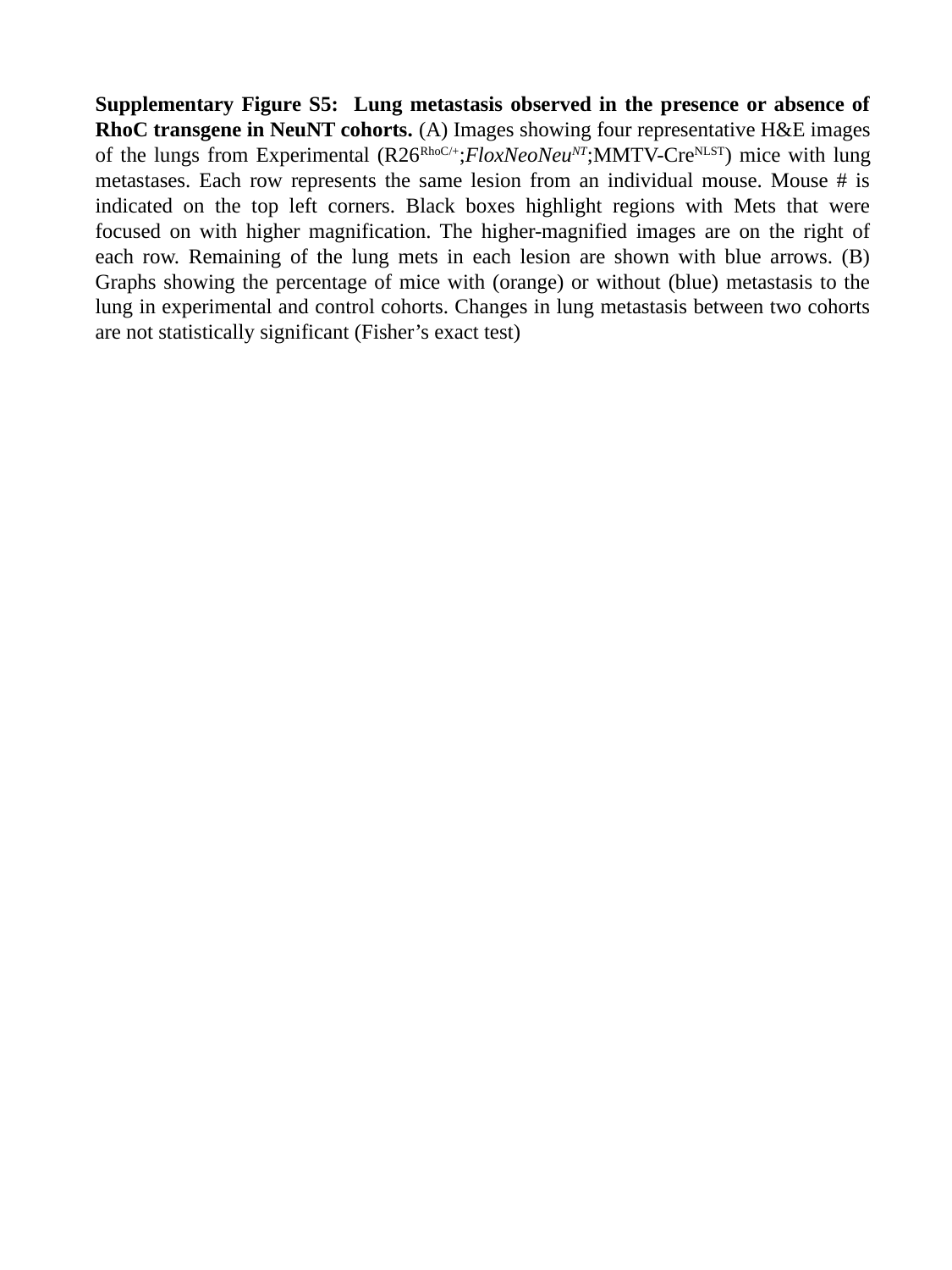

Supplementary Figure S5: Lung metastasis observed in the presence or absence of RhoC transgene in NeuNT cohorts. (A) Images showing four representative H&E images of the lungs from Experimental (R26RhoC/+;FloxNeoNeuNT;MMTV-CreNLST) mice with lung metastases. Each row represents the same lesion from an individual mouse. Mouse # is indicated on the top left corners. Black boxes highlight regions with Mets that were focused on with higher magnification. The higher-magnified images are on the right of each row. Remaining of the lung mets in each lesion are shown with blue arrows. (B) Graphs showing the percentage of mice with (orange) or without (blue) metastasis to the lung in experimental and control cohorts. Changes in lung metastasis between two cohorts are not statistically significant (Fisher’s exact test)

## Slide 12
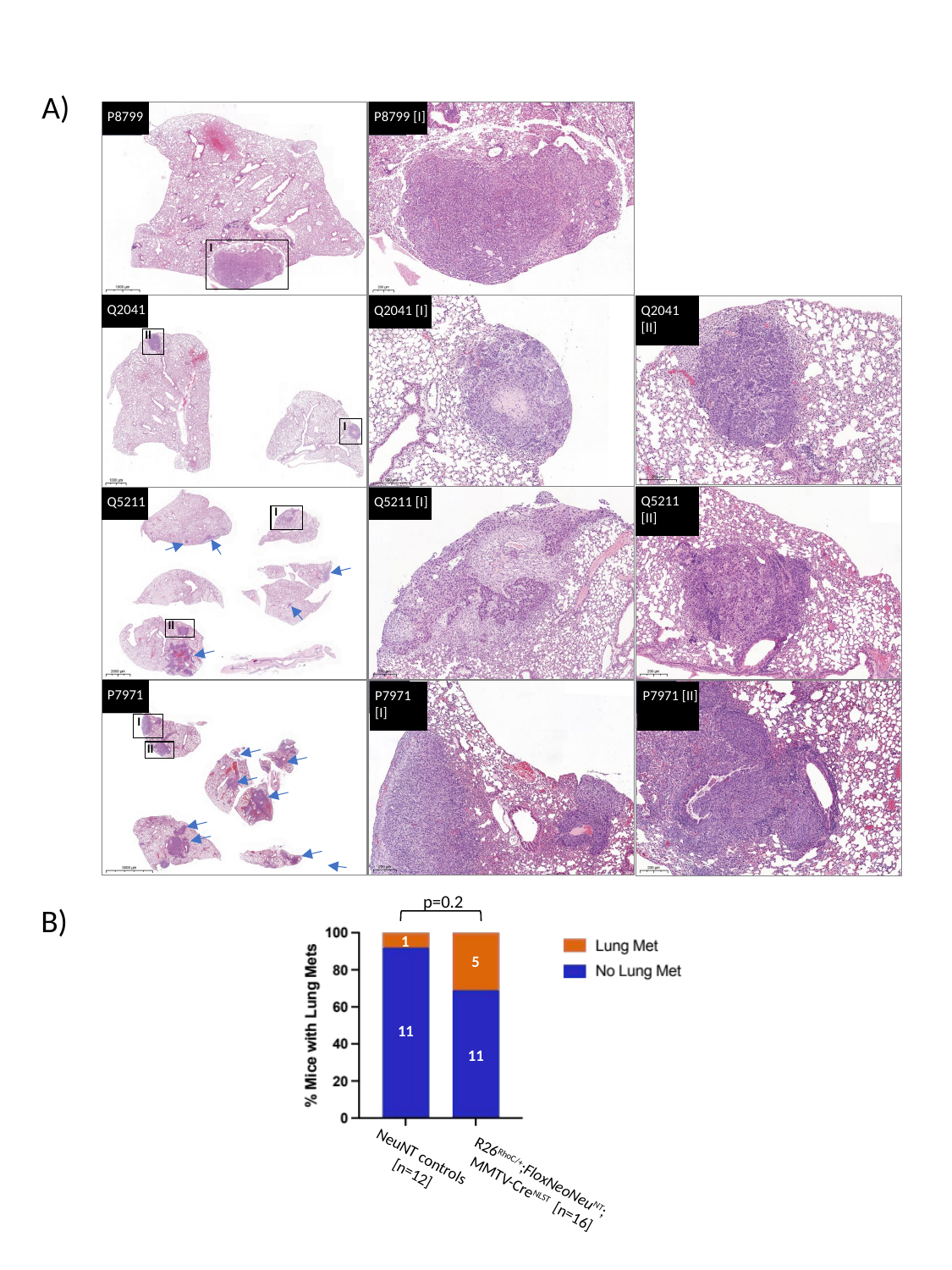

A)
P8799
P8799 [I]
I
Q2041
Q2041 [II]
Q2041 [I]
II
I
Q5211 [II]
Q5211 [I]
Q5211
I
II
P7971
P7971 [I]
P7971 [II]
I
II
p=0.2
1
5
11
11
B)
NeuNT controls
[n=12]
R26RhoC/+;FloxNeoNeuNT;
MMTV-CreNLST [n=16]

## Slide 13
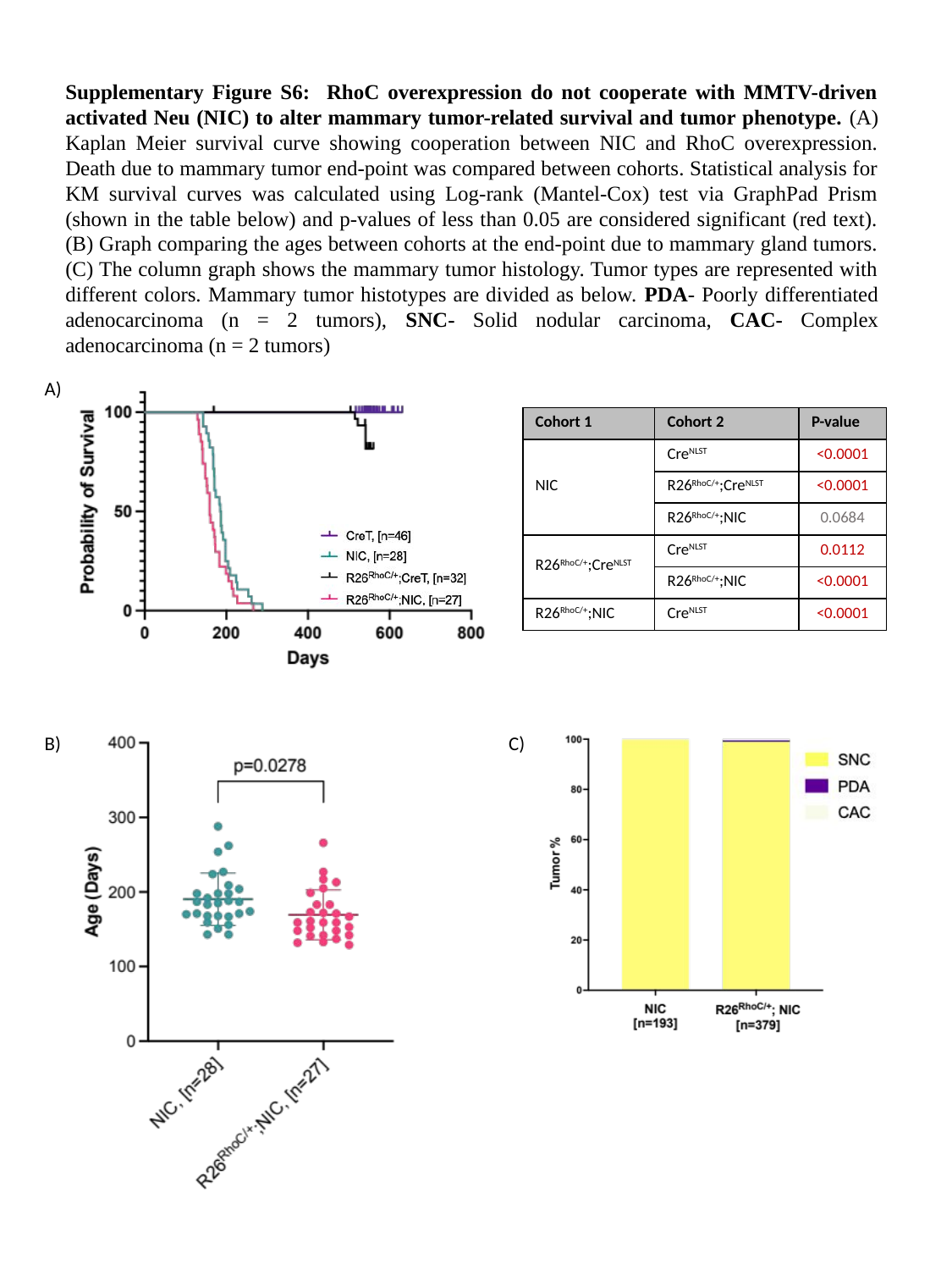

Supplementary Figure S6: RhoC overexpression do not cooperate with MMTV-driven activated Neu (NIC) to alter mammary tumor-related survival and tumor phenotype. (A) Kaplan Meier survival curve showing cooperation between NIC and RhoC overexpression. Death due to mammary tumor end-point was compared between cohorts. Statistical analysis for KM survival curves was calculated using Log-rank (Mantel-Cox) test via GraphPad Prism (shown in the table below) and p-values of less than 0.05 are considered significant (red text). (B) Graph comparing the ages between cohorts at the end-point due to mammary gland tumors. (C) The column graph shows the mammary tumor histology. Tumor types are represented with different colors. Mammary tumor histotypes are divided as below. PDA- Poorly differentiated adenocarcinoma (n = 2 tumors), SNC- Solid nodular carcinoma, CAC- Complex adenocarcinoma (n = 2 tumors)
A)
| Cohort 1 | Cohort 2 | P-value |
| --- | --- | --- |
| NIC | CreNLST | <0.0001 |
| | R26RhoC/+;CreNLST | <0.0001 |
| | R26RhoC/+;NIC | 0.0684 |
| R26RhoC/+;CreNLST | CreNLST | 0.0112 |
| | R26RhoC/+;NIC | <0.0001 |
| R26RhoC/+;NIC | CreNLST | <0.0001 |
B)
C)

## Slide 14
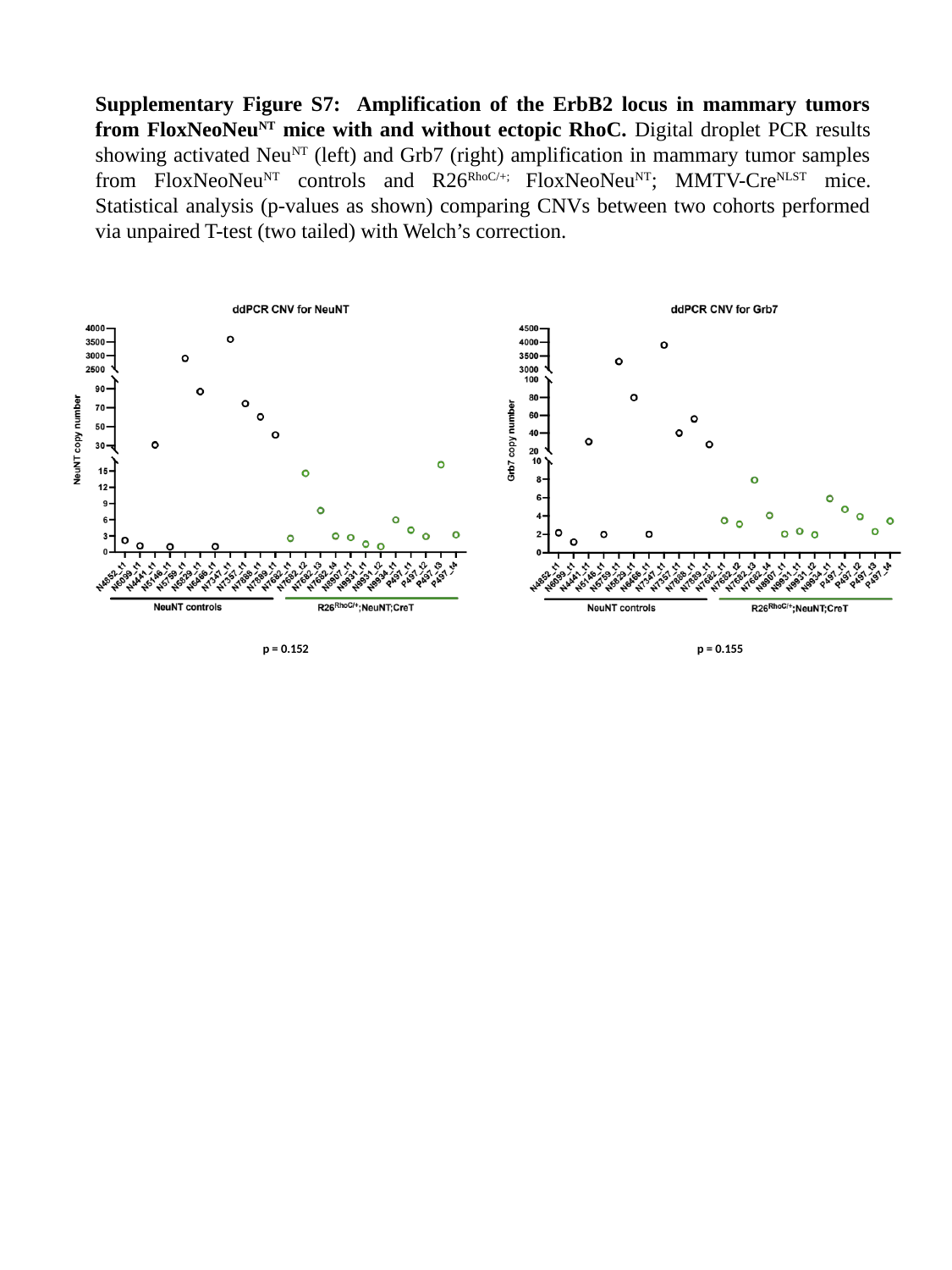

Supplementary Figure S7: Amplification of the ErbB2 locus in mammary tumors from FloxNeoNeuNT mice with and without ectopic RhoC. Digital droplet PCR results showing activated NeuNT (left) and Grb7 (right) amplification in mammary tumor samples from FloxNeoNeuNT controls and R26RhoC/+; FloxNeoNeuNT; MMTV-CreNLST mice. Statistical analysis (p-values as shown) comparing CNVs between two cohorts performed via unpaired T-test (two tailed) with Welch’s correction.
p = 0.152
p = 0.155

## Slide 15
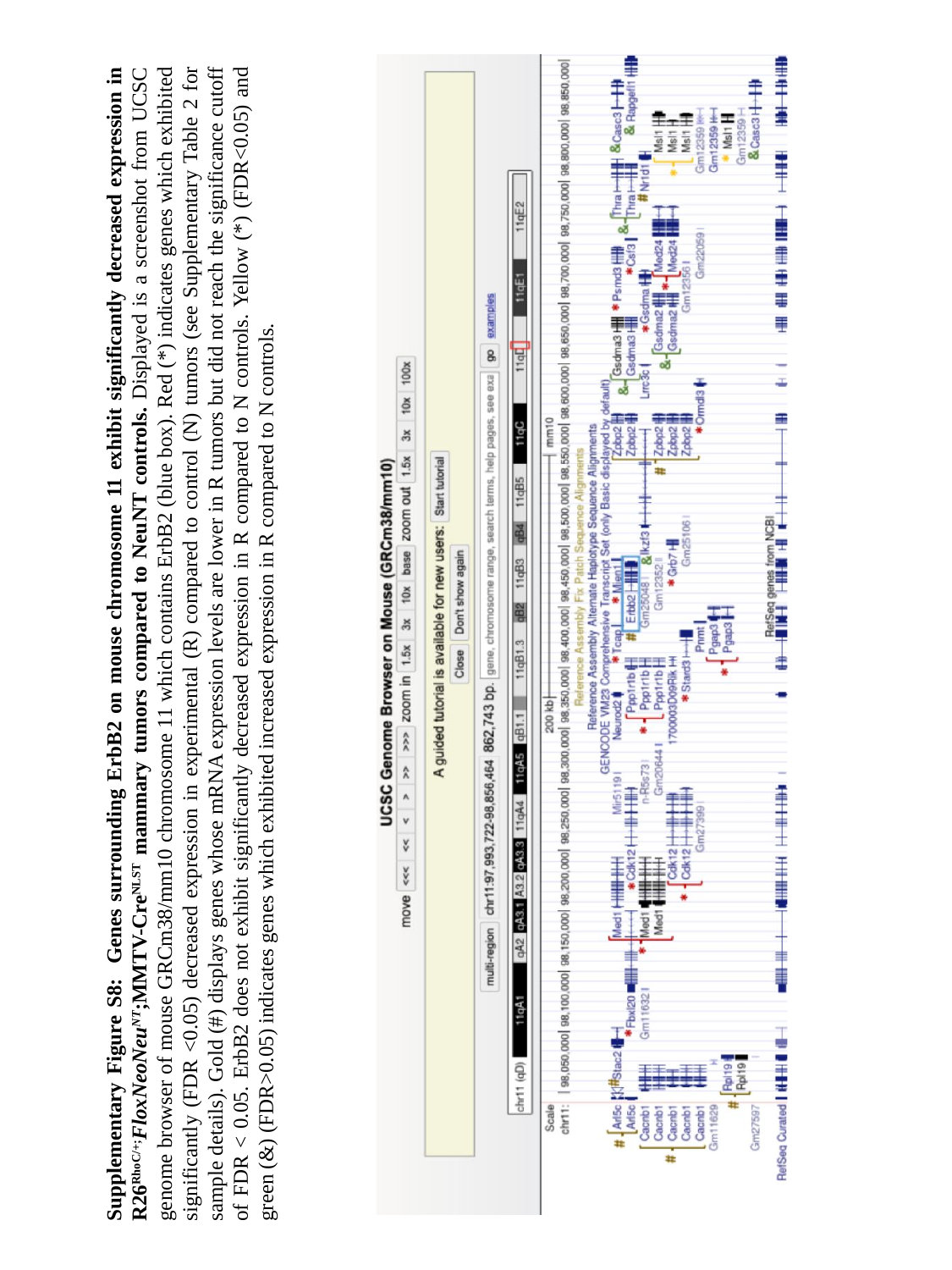

Supplementary Figure S8: Genes surrounding ErbB2 on mouse chromosome 11 exhibit significantly decreased expression in R26RhoC/+;FloxNeoNeuNT;MMTV-CreNLST mammary tumors compared to NeuNT controls. Displayed is a screenshot from UCSC genome browser of mouse GRCm38/mm10 chromosome 11 which contains ErbB2 (blue box). Red (*) indicates genes which exhibited significantly (FDR <0.05) decreased expression in experimental (R) compared to control (N) tumors (see Supplementary Table 2 for sample details). Gold (#) displays genes whose mRNA expression levels are lower in R tumors but did not reach the significance cutoff of FDR < 0.05. ErbB2 does not exhibit significantly decreased expression in R compared to N controls. Yellow (*) (FDR<0.05) and green (&) (FDR>0.05) indicates genes which exhibited increased expression in R compared to N controls.

## Slide 16
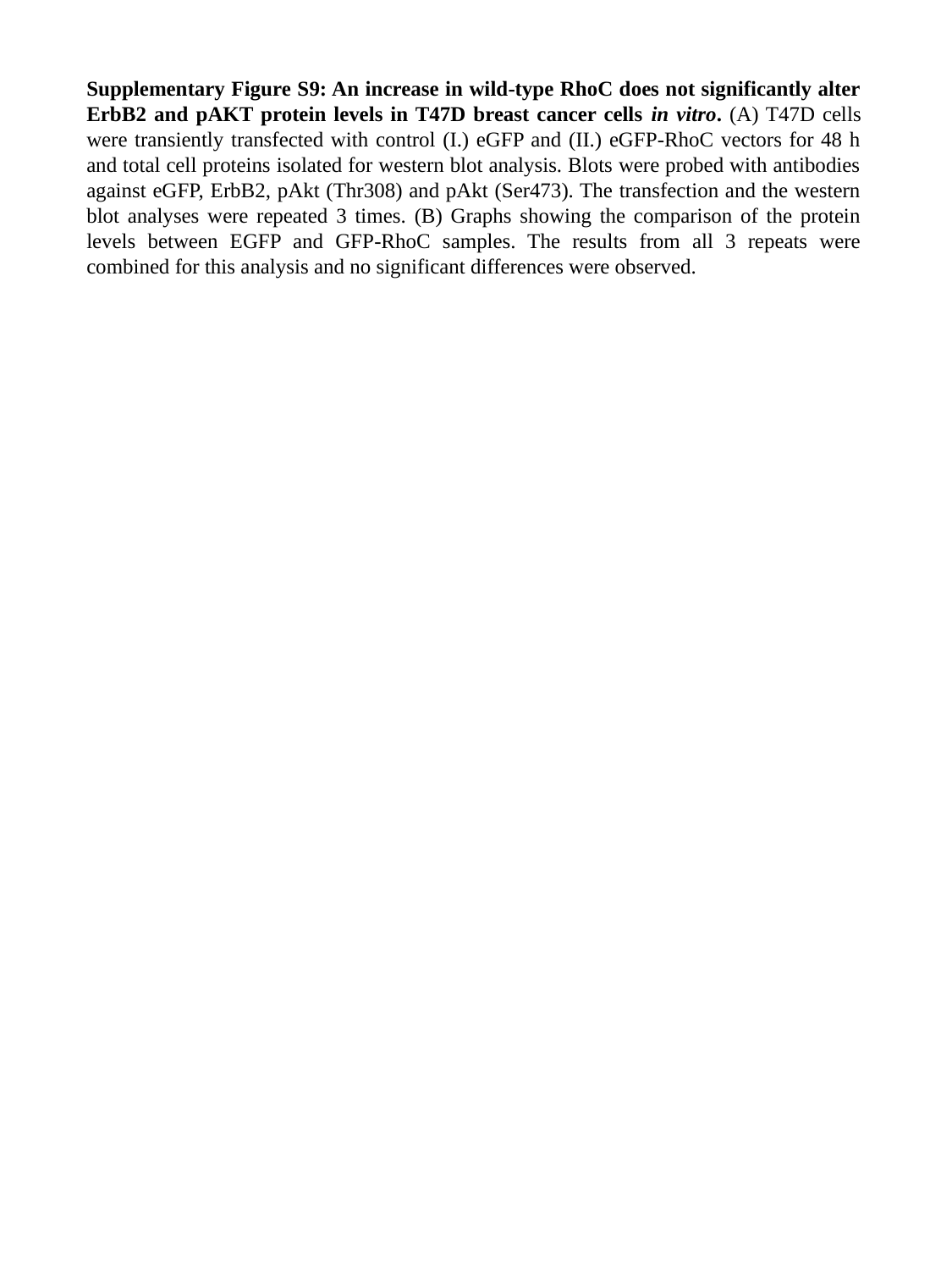

Supplementary Figure S9: An increase in wild-type RhoC does not significantly alter ErbB2 and pAKT protein levels in T47D breast cancer cells in vitro. (A) T47D cells were transiently transfected with control (I.) eGFP and (II.) eGFP-RhoC vectors for 48 h and total cell proteins isolated for western blot analysis. Blots were probed with antibodies against eGFP, ErbB2, pAkt (Thr308) and pAkt (Ser473). The transfection and the western blot analyses were repeated 3 times. (B) Graphs showing the comparison of the protein levels between EGFP and GFP-RhoC samples. The results from all 3 repeats were combined for this analysis and no significant differences were observed.

## Slide 17
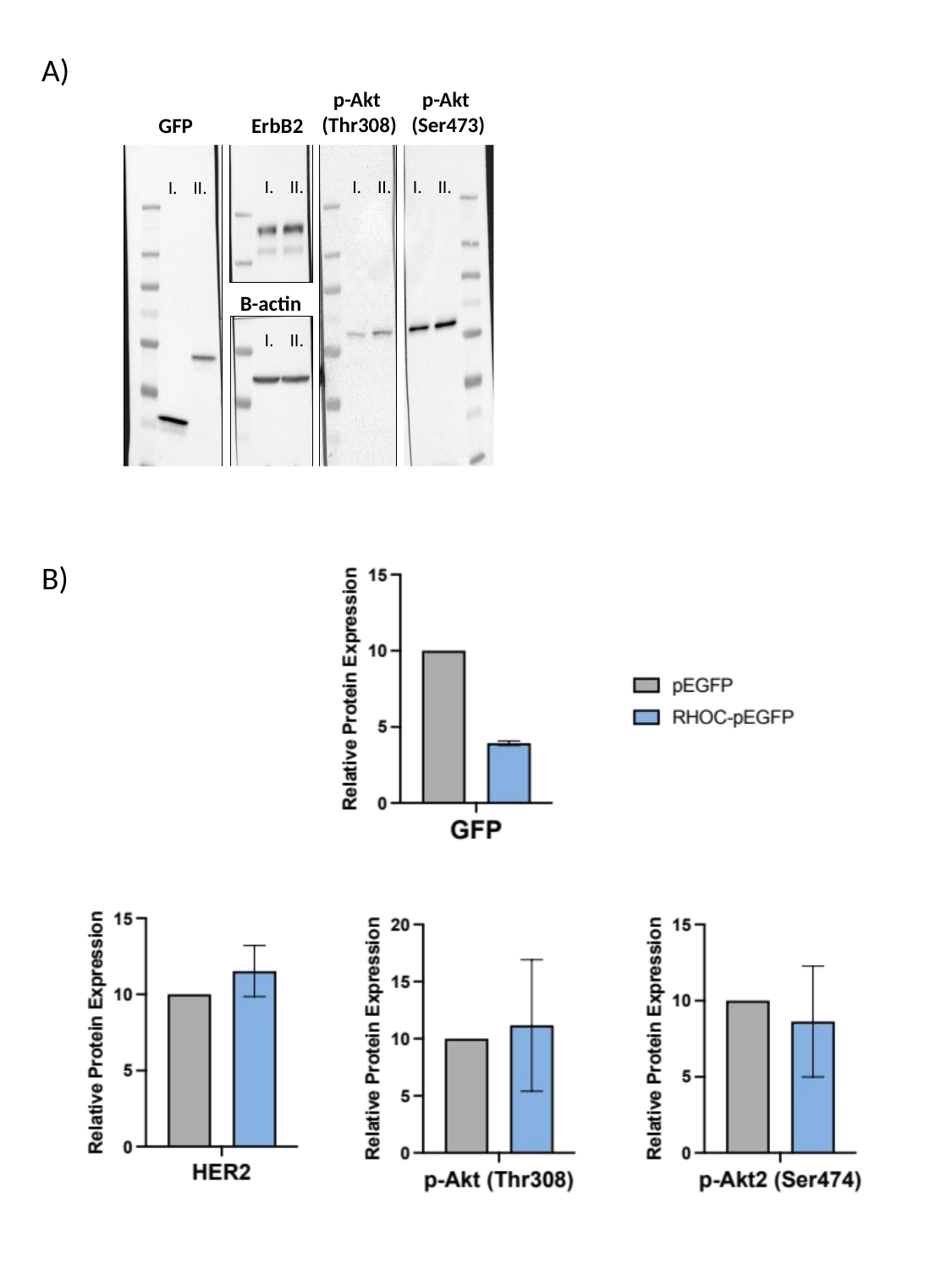

A)
p-Akt
(Thr308)
p-Akt
(Ser473)
GFP
ErbB2
I.
II.
I.
II.
I.
II.
I.
II.
B-actin
I.
II.
B)

## Slide 18
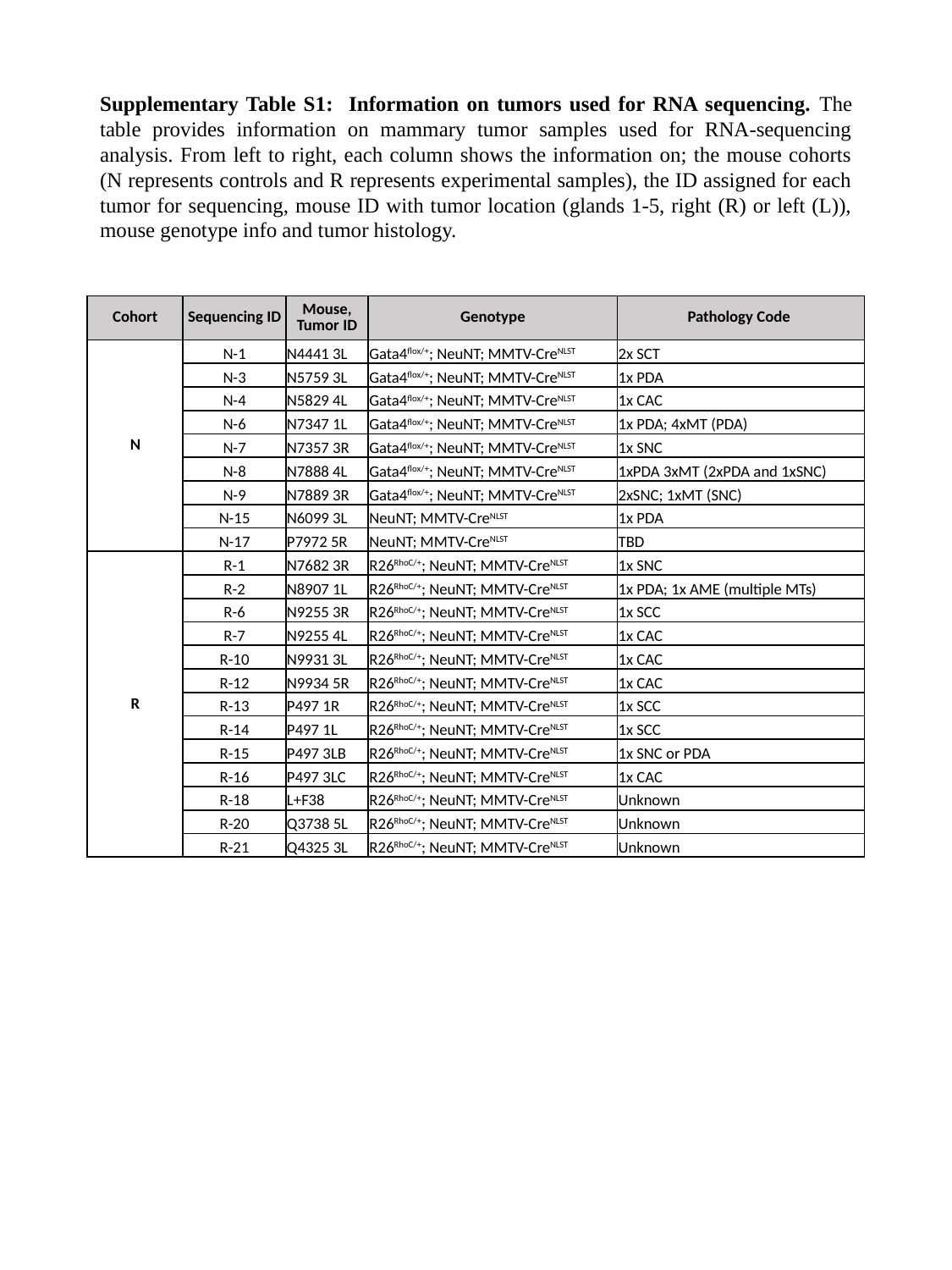

Supplementary Table S1: Information on tumors used for RNA sequencing. The table provides information on mammary tumor samples used for RNA-sequencing analysis. From left to right, each column shows the information on; the mouse cohorts (N represents controls and R represents experimental samples), the ID assigned for each tumor for sequencing, mouse ID with tumor location (glands 1-5, right (R) or left (L)), mouse genotype info and tumor histology.
| Cohort | Sequencing ID | Mouse, Tumor ID | Genotype | Pathology Code |
| --- | --- | --- | --- | --- |
| N | N-1 | N4441 3L | Gata4flox/+; NeuNT; MMTV-CreNLST | 2x SCT |
| | N-3 | N5759 3L | Gata4flox/+; NeuNT; MMTV-CreNLST | 1x PDA |
| | N-4 | N5829 4L | Gata4flox/+; NeuNT; MMTV-CreNLST | 1x CAC |
| | N-6 | N7347 1L | Gata4flox/+; NeuNT; MMTV-CreNLST | 1x PDA; 4xMT (PDA) |
| | N-7 | N7357 3R | Gata4flox/+; NeuNT; MMTV-CreNLST | 1x SNC |
| | N-8 | N7888 4L | Gata4flox/+; NeuNT; MMTV-CreNLST | 1xPDA 3xMT (2xPDA and 1xSNC) |
| | N-9 | N7889 3R | Gata4flox/+; NeuNT; MMTV-CreNLST | 2xSNC; 1xMT (SNC) |
| | N-15 | N6099 3L | NeuNT; MMTV-CreNLST | 1x PDA |
| | N-17 | P7972 5R | NeuNT; MMTV-CreNLST | TBD |
| R | R-1 | N7682 3R | R26RhoC/+; NeuNT; MMTV-CreNLST | 1x SNC |
| | R-2 | N8907 1L | R26RhoC/+; NeuNT; MMTV-CreNLST | 1x PDA; 1x AME (multiple MTs) |
| | R-6 | N9255 3R | R26RhoC/+; NeuNT; MMTV-CreNLST | 1x SCC |
| | R-7 | N9255 4L | R26RhoC/+; NeuNT; MMTV-CreNLST | 1x CAC |
| | R-10 | N9931 3L | R26RhoC/+; NeuNT; MMTV-CreNLST | 1x CAC |
| | R-12 | N9934 5R | R26RhoC/+; NeuNT; MMTV-CreNLST | 1x CAC |
| | R-13 | P497 1R | R26RhoC/+; NeuNT; MMTV-CreNLST | 1x SCC |
| | R-14 | P497 1L | R26RhoC/+; NeuNT; MMTV-CreNLST | 1x SCC |
| | R-15 | P497 3LB | R26RhoC/+; NeuNT; MMTV-CreNLST | 1x SNC or PDA |
| | R-16 | P497 3LC | R26RhoC/+; NeuNT; MMTV-CreNLST | 1x CAC |
| | R-18 | L+F38 | R26RhoC/+; NeuNT; MMTV-CreNLST | Unknown |
| | R-20 | Q3738 5L | R26RhoC/+; NeuNT; MMTV-CreNLST | Unknown |
| | R-21 | Q4325 3L | R26RhoC/+; NeuNT; MMTV-CreNLST | Unknown |

## Slide 19
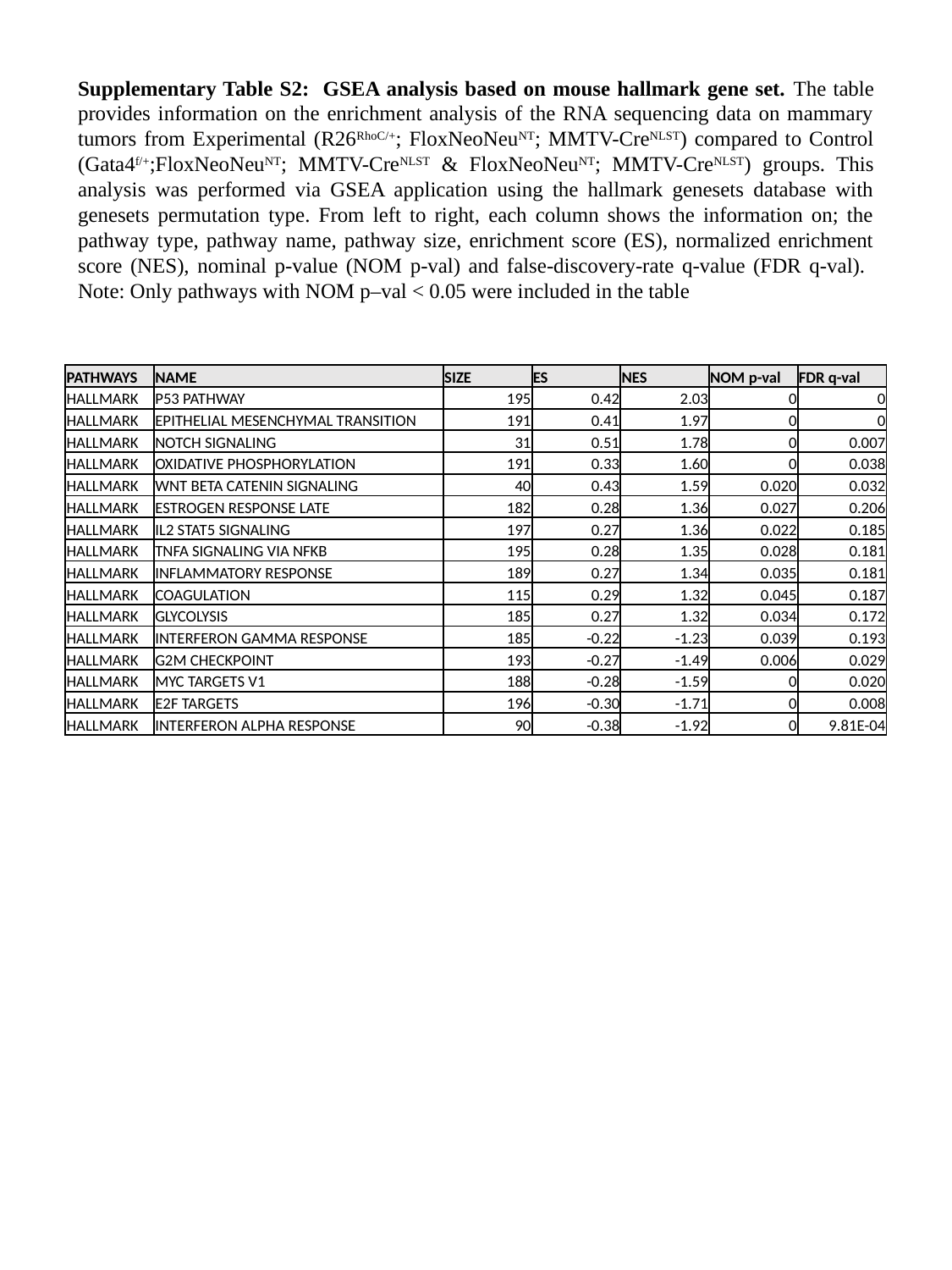

Supplementary Table S2: GSEA analysis based on mouse hallmark gene set. The table provides information on the enrichment analysis of the RNA sequencing data on mammary tumors from Experimental (R26RhoC/+; FloxNeoNeuNT; MMTV-CreNLST) compared to Control (Gata4f/+;FloxNeoNeuNT; MMTV-CreNLST & FloxNeoNeuNT; MMTV-CreNLST) groups. This analysis was performed via GSEA application using the hallmark genesets database with genesets permutation type. From left to right, each column shows the information on; the pathway type, pathway name, pathway size, enrichment score (ES), normalized enrichment score (NES), nominal p-value (NOM p-val) and false-discovery-rate q-value (FDR q-val). Note: Only pathways with NOM p–val < 0.05 were included in the table
| PATHWAYS | NAME | SIZE | ES | NES | NOM p-val | FDR q-val |
| --- | --- | --- | --- | --- | --- | --- |
| HALLMARK | P53 PATHWAY | 195 | 0.42 | 2.03 | 0 | 0 |
| HALLMARK | EPITHELIAL MESENCHYMAL TRANSITION | 191 | 0.41 | 1.97 | 0 | 0 |
| HALLMARK | NOTCH SIGNALING | 31 | 0.51 | 1.78 | 0 | 0.007 |
| HALLMARK | OXIDATIVE PHOSPHORYLATION | 191 | 0.33 | 1.60 | 0 | 0.038 |
| HALLMARK | WNT BETA CATENIN SIGNALING | 40 | 0.43 | 1.59 | 0.020 | 0.032 |
| HALLMARK | ESTROGEN RESPONSE LATE | 182 | 0.28 | 1.36 | 0.027 | 0.206 |
| HALLMARK | IL2 STAT5 SIGNALING | 197 | 0.27 | 1.36 | 0.022 | 0.185 |
| HALLMARK | TNFA SIGNALING VIA NFKB | 195 | 0.28 | 1.35 | 0.028 | 0.181 |
| HALLMARK | INFLAMMATORY RESPONSE | 189 | 0.27 | 1.34 | 0.035 | 0.181 |
| HALLMARK | COAGULATION | 115 | 0.29 | 1.32 | 0.045 | 0.187 |
| HALLMARK | GLYCOLYSIS | 185 | 0.27 | 1.32 | 0.034 | 0.172 |
| HALLMARK | INTERFERON GAMMA RESPONSE | 185 | -0.22 | -1.23 | 0.039 | 0.193 |
| HALLMARK | G2M CHECKPOINT | 193 | -0.27 | -1.49 | 0.006 | 0.029 |
| HALLMARK | MYC TARGETS V1 | 188 | -0.28 | -1.59 | 0 | 0.020 |
| HALLMARK | E2F TARGETS | 196 | -0.30 | -1.71 | 0 | 0.008 |
| HALLMARK | INTERFERON ALPHA RESPONSE | 90 | -0.38 | -1.92 | 0 | 9.81E-04 |

## Slide 20
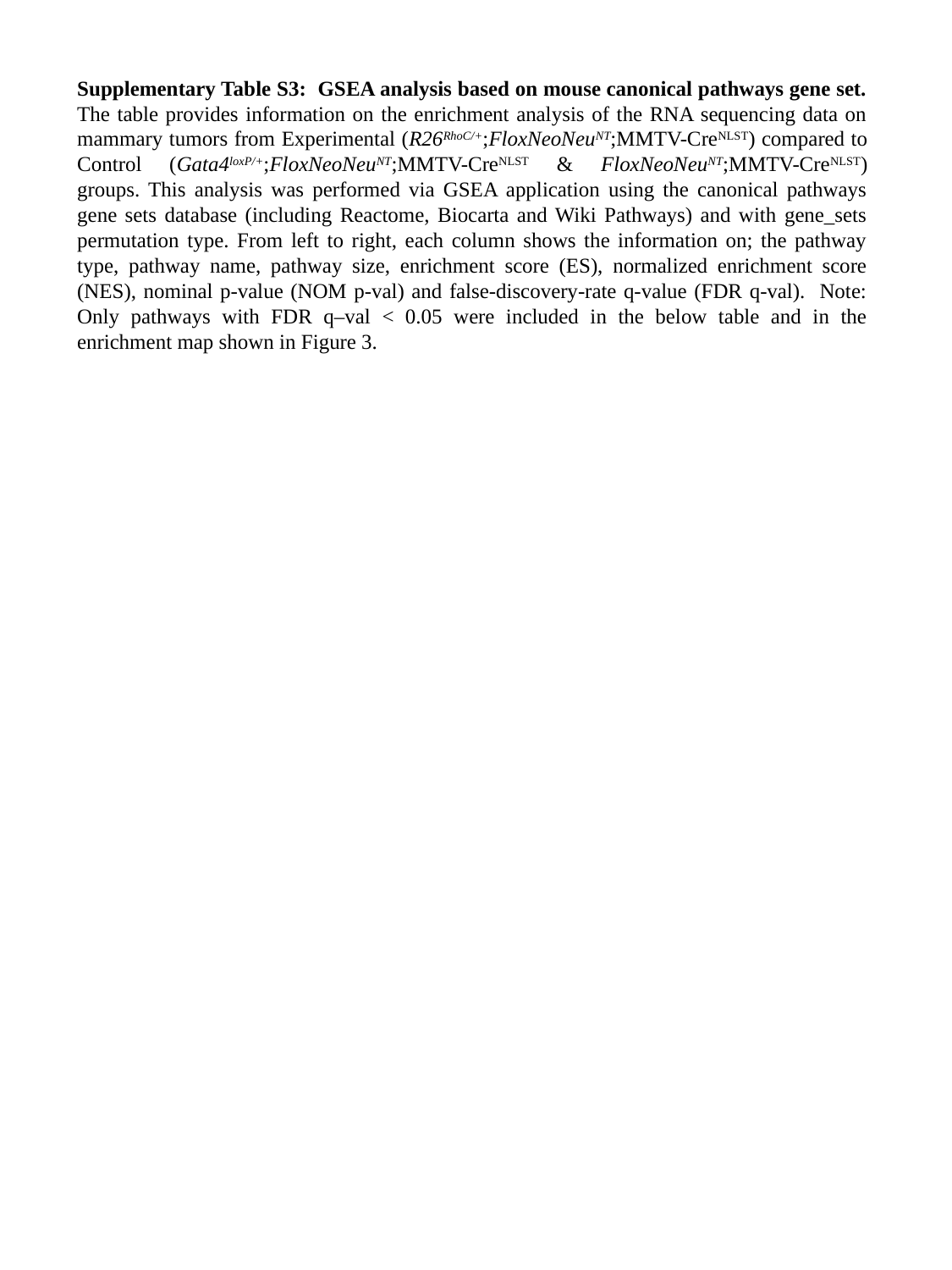

Supplementary Table S3: GSEA analysis based on mouse canonical pathways gene set. The table provides information on the enrichment analysis of the RNA sequencing data on mammary tumors from Experimental (R26RhoC/+;FloxNeoNeuNT;MMTV-CreNLST) compared to Control (Gata4loxP/+;FloxNeoNeuNT;MMTV-CreNLST & FloxNeoNeuNT;MMTV-CreNLST) groups. This analysis was performed via GSEA application using the canonical pathways gene sets database (including Reactome, Biocarta and Wiki Pathways) and with gene_sets permutation type. From left to right, each column shows the information on; the pathway type, pathway name, pathway size, enrichment score (ES), normalized enrichment score (NES), nominal p-value (NOM p-val) and false-discovery-rate q-value (FDR q-val). Note: Only pathways with FDR q–val < 0.05 were included in the below table and in the enrichment map shown in Figure 3.

## Slide 21
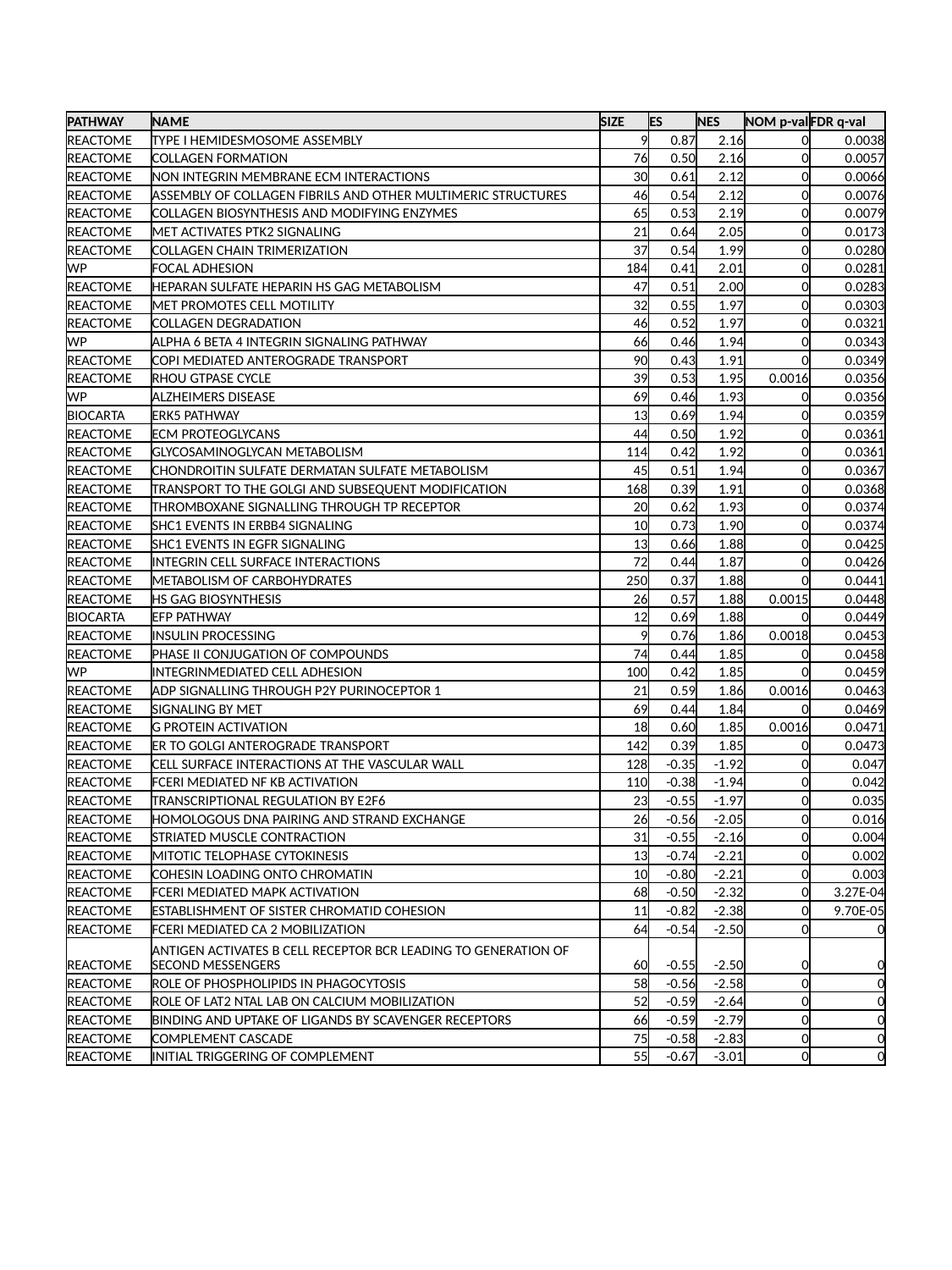

| PATHWAY | NAME | SIZE | ES | NES | NOM p-val | FDR q-val |
| --- | --- | --- | --- | --- | --- | --- |
| REACTOME | TYPE I HEMIDESMOSOME ASSEMBLY | 9 | 0.87 | 2.16 | 0 | 0.0038 |
| REACTOME | COLLAGEN FORMATION | 76 | 0.50 | 2.16 | 0 | 0.0057 |
| REACTOME | NON INTEGRIN MEMBRANE ECM INTERACTIONS | 30 | 0.61 | 2.12 | 0 | 0.0066 |
| REACTOME | ASSEMBLY OF COLLAGEN FIBRILS AND OTHER MULTIMERIC STRUCTURES | 46 | 0.54 | 2.12 | 0 | 0.0076 |
| REACTOME | COLLAGEN BIOSYNTHESIS AND MODIFYING ENZYMES | 65 | 0.53 | 2.19 | 0 | 0.0079 |
| REACTOME | MET ACTIVATES PTK2 SIGNALING | 21 | 0.64 | 2.05 | 0 | 0.0173 |
| REACTOME | COLLAGEN CHAIN TRIMERIZATION | 37 | 0.54 | 1.99 | 0 | 0.0280 |
| WP | FOCAL ADHESION | 184 | 0.41 | 2.01 | 0 | 0.0281 |
| REACTOME | HEPARAN SULFATE HEPARIN HS GAG METABOLISM | 47 | 0.51 | 2.00 | 0 | 0.0283 |
| REACTOME | MET PROMOTES CELL MOTILITY | 32 | 0.55 | 1.97 | 0 | 0.0303 |
| REACTOME | COLLAGEN DEGRADATION | 46 | 0.52 | 1.97 | 0 | 0.0321 |
| WP | ALPHA 6 BETA 4 INTEGRIN SIGNALING PATHWAY | 66 | 0.46 | 1.94 | 0 | 0.0343 |
| REACTOME | COPI MEDIATED ANTEROGRADE TRANSPORT | 90 | 0.43 | 1.91 | 0 | 0.0349 |
| REACTOME | RHOU GTPASE CYCLE | 39 | 0.53 | 1.95 | 0.0016 | 0.0356 |
| WP | ALZHEIMERS DISEASE | 69 | 0.46 | 1.93 | 0 | 0.0356 |
| BIOCARTA | ERK5 PATHWAY | 13 | 0.69 | 1.94 | 0 | 0.0359 |
| REACTOME | ECM PROTEOGLYCANS | 44 | 0.50 | 1.92 | 0 | 0.0361 |
| REACTOME | GLYCOSAMINOGLYCAN METABOLISM | 114 | 0.42 | 1.92 | 0 | 0.0361 |
| REACTOME | CHONDROITIN SULFATE DERMATAN SULFATE METABOLISM | 45 | 0.51 | 1.94 | 0 | 0.0367 |
| REACTOME | TRANSPORT TO THE GOLGI AND SUBSEQUENT MODIFICATION | 168 | 0.39 | 1.91 | 0 | 0.0368 |
| REACTOME | THROMBOXANE SIGNALLING THROUGH TP RECEPTOR | 20 | 0.62 | 1.93 | 0 | 0.0374 |
| REACTOME | SHC1 EVENTS IN ERBB4 SIGNALING | 10 | 0.73 | 1.90 | 0 | 0.0374 |
| REACTOME | SHC1 EVENTS IN EGFR SIGNALING | 13 | 0.66 | 1.88 | 0 | 0.0425 |
| REACTOME | INTEGRIN CELL SURFACE INTERACTIONS | 72 | 0.44 | 1.87 | 0 | 0.0426 |
| REACTOME | METABOLISM OF CARBOHYDRATES | 250 | 0.37 | 1.88 | 0 | 0.0441 |
| REACTOME | HS GAG BIOSYNTHESIS | 26 | 0.57 | 1.88 | 0.0015 | 0.0448 |
| BIOCARTA | EFP PATHWAY | 12 | 0.69 | 1.88 | 0 | 0.0449 |
| REACTOME | INSULIN PROCESSING | 9 | 0.76 | 1.86 | 0.0018 | 0.0453 |
| REACTOME | PHASE II CONJUGATION OF COMPOUNDS | 74 | 0.44 | 1.85 | 0 | 0.0458 |
| WP | INTEGRINMEDIATED CELL ADHESION | 100 | 0.42 | 1.85 | 0 | 0.0459 |
| REACTOME | ADP SIGNALLING THROUGH P2Y PURINOCEPTOR 1 | 21 | 0.59 | 1.86 | 0.0016 | 0.0463 |
| REACTOME | SIGNALING BY MET | 69 | 0.44 | 1.84 | 0 | 0.0469 |
| REACTOME | G PROTEIN ACTIVATION | 18 | 0.60 | 1.85 | 0.0016 | 0.0471 |
| REACTOME | ER TO GOLGI ANTEROGRADE TRANSPORT | 142 | 0.39 | 1.85 | 0 | 0.0473 |
| REACTOME | CELL SURFACE INTERACTIONS AT THE VASCULAR WALL | 128 | -0.35 | -1.92 | 0 | 0.047 |
| REACTOME | FCERI MEDIATED NF KB ACTIVATION | 110 | -0.38 | -1.94 | 0 | 0.042 |
| REACTOME | TRANSCRIPTIONAL REGULATION BY E2F6 | 23 | -0.55 | -1.97 | 0 | 0.035 |
| REACTOME | HOMOLOGOUS DNA PAIRING AND STRAND EXCHANGE | 26 | -0.56 | -2.05 | 0 | 0.016 |
| REACTOME | STRIATED MUSCLE CONTRACTION | 31 | -0.55 | -2.16 | 0 | 0.004 |
| REACTOME | MITOTIC TELOPHASE CYTOKINESIS | 13 | -0.74 | -2.21 | 0 | 0.002 |
| REACTOME | COHESIN LOADING ONTO CHROMATIN | 10 | -0.80 | -2.21 | 0 | 0.003 |
| REACTOME | FCERI MEDIATED MAPK ACTIVATION | 68 | -0.50 | -2.32 | 0 | 3.27E-04 |
| REACTOME | ESTABLISHMENT OF SISTER CHROMATID COHESION | 11 | -0.82 | -2.38 | 0 | 9.70E-05 |
| REACTOME | FCERI MEDIATED CA 2 MOBILIZATION | 64 | -0.54 | -2.50 | 0 | 0 |
| REACTOME | ANTIGEN ACTIVATES B CELL RECEPTOR BCR LEADING TO GENERATION OF SECOND MESSENGERS | 60 | -0.55 | -2.50 | 0 | 0 |
| REACTOME | ROLE OF PHOSPHOLIPIDS IN PHAGOCYTOSIS | 58 | -0.56 | -2.58 | 0 | 0 |
| REACTOME | ROLE OF LAT2 NTAL LAB ON CALCIUM MOBILIZATION | 52 | -0.59 | -2.64 | 0 | 0 |
| REACTOME | BINDING AND UPTAKE OF LIGANDS BY SCAVENGER RECEPTORS | 66 | -0.59 | -2.79 | 0 | 0 |
| REACTOME | COMPLEMENT CASCADE | 75 | -0.58 | -2.83 | 0 | 0 |
| REACTOME | INITIAL TRIGGERING OF COMPLEMENT | 55 | -0.67 | -3.01 | 0 | 0 |

## Slide 22
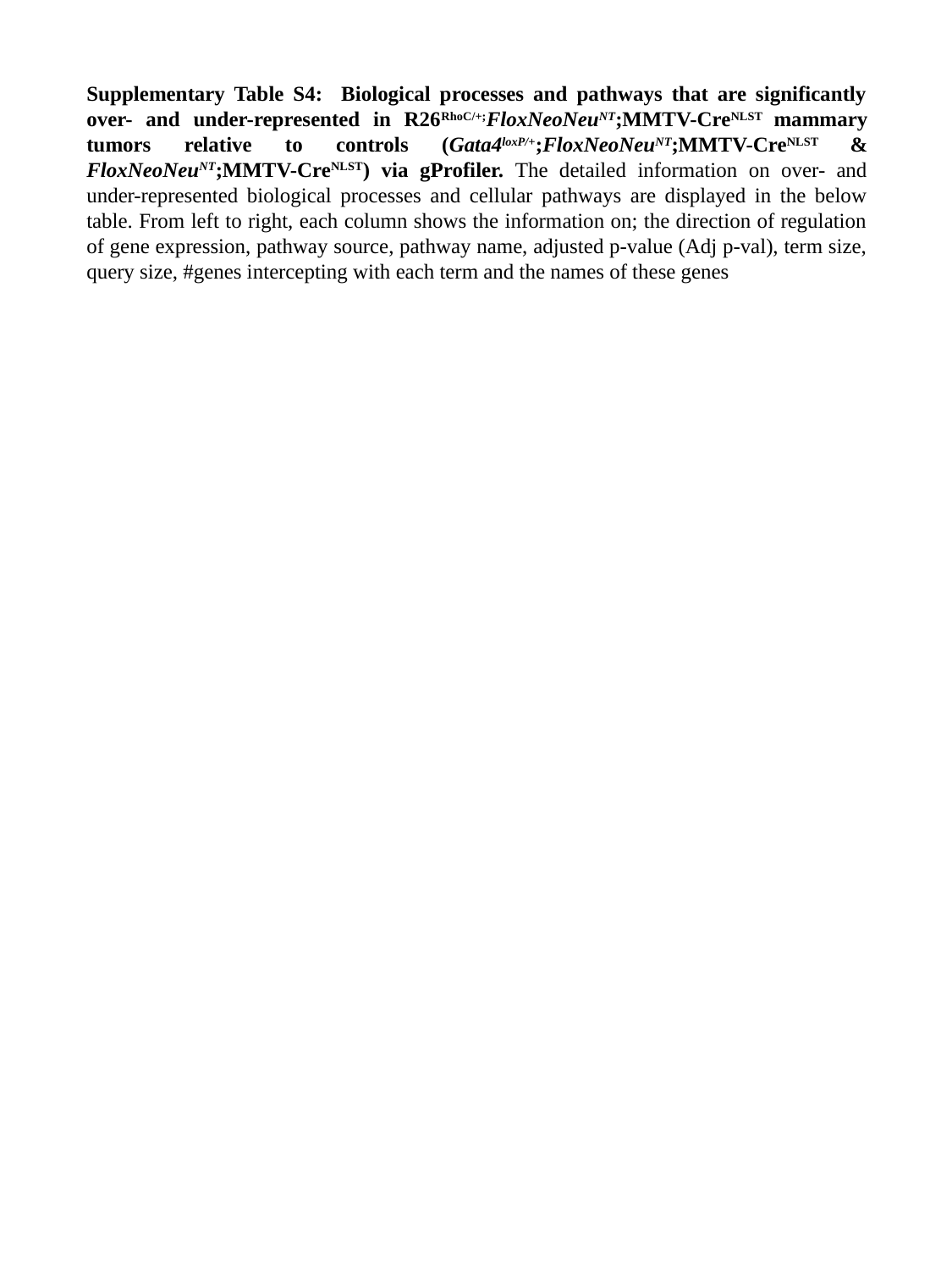

Supplementary Table S4: Biological processes and pathways that are significantly over- and under-represented in R26RhoC/+;FloxNeoNeuNT;MMTV-CreNLST mammary tumors relative to controls (Gata4loxP/+;FloxNeoNeuNT;MMTV-CreNLST & FloxNeoNeuNT;MMTV-CreNLST) via gProfiler. The detailed information on over- and under-represented biological processes and cellular pathways are displayed in the below table. From left to right, each column shows the information on; the direction of regulation of gene expression, pathway source, pathway name, adjusted p-value (Adj p-val), term size, query size, #genes intercepting with each term and the names of these genes

## Slide 23
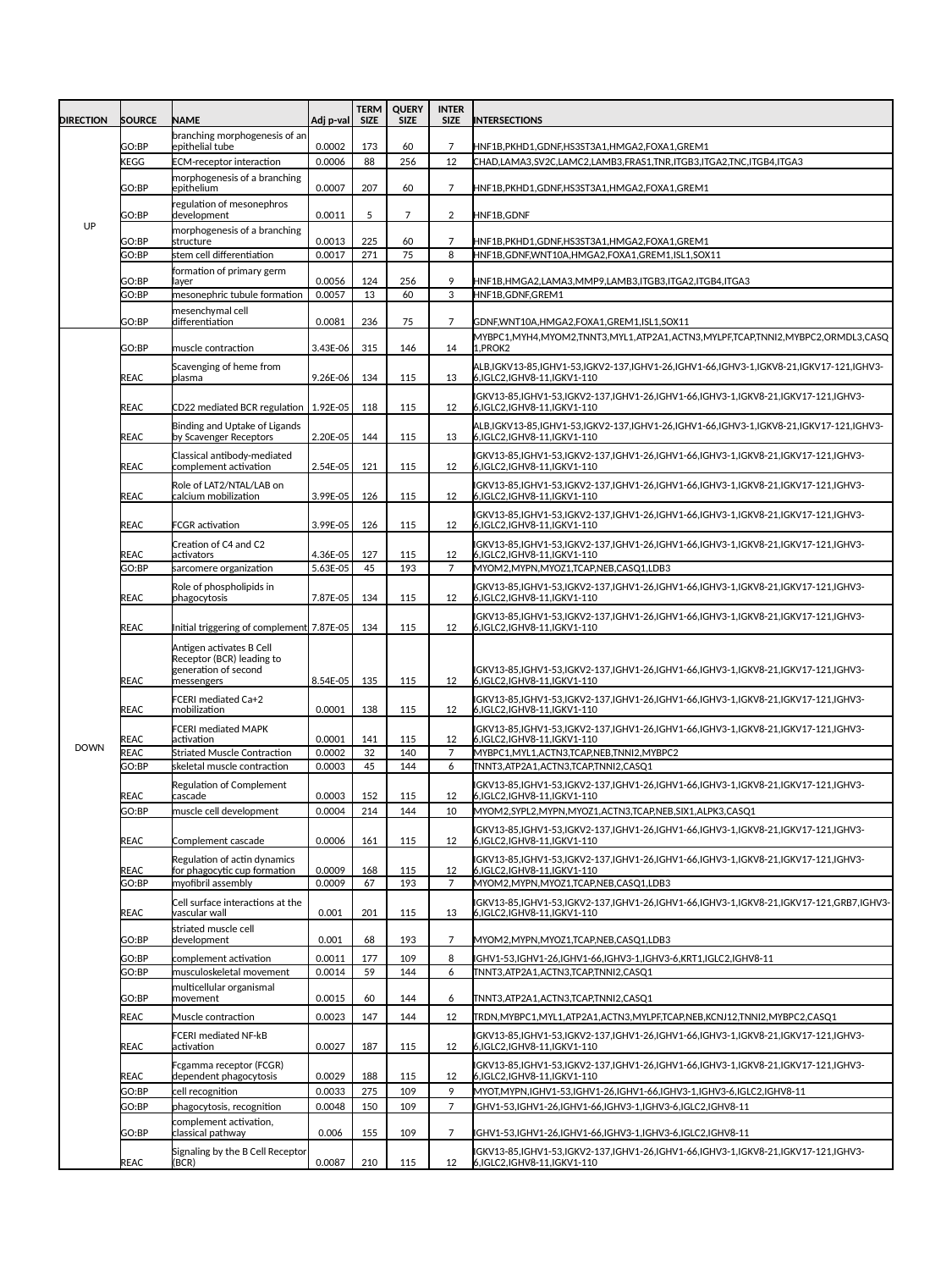

| DIRECTION | SOURCE | NAME | Adj p-val | TERM SIZE | QUERY SIZE | INTER SIZE | INTERSECTIONS |
| --- | --- | --- | --- | --- | --- | --- | --- |
| UP | GO:BP | branching morphogenesis of an epithelial tube | 0.0002 | 173 | 60 | 7 | HNF1B,PKHD1,GDNF,HS3ST3A1,HMGA2,FOXA1,GREM1 |
| | KEGG | ECM-receptor interaction | 0.0006 | 88 | 256 | 12 | CHAD,LAMA3,SV2C,LAMC2,LAMB3,FRAS1,TNR,ITGB3,ITGA2,TNC,ITGB4,ITGA3 |
| | GO:BP | morphogenesis of a branching epithelium | 0.0007 | 207 | 60 | 7 | HNF1B,PKHD1,GDNF,HS3ST3A1,HMGA2,FOXA1,GREM1 |
| | GO:BP | regulation of mesonephros development | 0.0011 | 5 | 7 | 2 | HNF1B,GDNF |
| | GO:BP | morphogenesis of a branching structure | 0.0013 | 225 | 60 | 7 | HNF1B,PKHD1,GDNF,HS3ST3A1,HMGA2,FOXA1,GREM1 |
| | GO:BP | stem cell differentiation | 0.0017 | 271 | 75 | 8 | HNF1B,GDNF,WNT10A,HMGA2,FOXA1,GREM1,ISL1,SOX11 |
| | GO:BP | formation of primary germ layer | 0.0056 | 124 | 256 | 9 | HNF1B,HMGA2,LAMA3,MMP9,LAMB3,ITGB3,ITGA2,ITGB4,ITGA3 |
| | GO:BP | mesonephric tubule formation | 0.0057 | 13 | 60 | 3 | HNF1B,GDNF,GREM1 |
| | GO:BP | mesenchymal cell differentiation | 0.0081 | 236 | 75 | 7 | GDNF,WNT10A,HMGA2,FOXA1,GREM1,ISL1,SOX11 |
| DOWN | GO:BP | muscle contraction | 3.43E-06 | 315 | 146 | 14 | MYBPC1,MYH4,MYOM2,TNNT3,MYL1,ATP2A1,ACTN3,MYLPF,TCAP,TNNI2,MYBPC2,ORMDL3,CASQ1,PROK2 |
| | REAC | Scavenging of heme from plasma | 9.26E-06 | 134 | 115 | 13 | ALB,IGKV13-85,IGHV1-53,IGKV2-137,IGHV1-26,IGHV1-66,IGHV3-1,IGKV8-21,IGKV17-121,IGHV3-6,IGLC2,IGHV8-11,IGKV1-110 |
| | REAC | CD22 mediated BCR regulation | 1.92E-05 | 118 | 115 | 12 | IGKV13-85,IGHV1-53,IGKV2-137,IGHV1-26,IGHV1-66,IGHV3-1,IGKV8-21,IGKV17-121,IGHV3-6,IGLC2,IGHV8-11,IGKV1-110 |
| | REAC | Binding and Uptake of Ligands by Scavenger Receptors | 2.20E-05 | 144 | 115 | 13 | ALB,IGKV13-85,IGHV1-53,IGKV2-137,IGHV1-26,IGHV1-66,IGHV3-1,IGKV8-21,IGKV17-121,IGHV3-6,IGLC2,IGHV8-11,IGKV1-110 |
| | REAC | Classical antibody-mediated complement activation | 2.54E-05 | 121 | 115 | 12 | IGKV13-85,IGHV1-53,IGKV2-137,IGHV1-26,IGHV1-66,IGHV3-1,IGKV8-21,IGKV17-121,IGHV3-6,IGLC2,IGHV8-11,IGKV1-110 |
| | REAC | Role of LAT2/NTAL/LAB on calcium mobilization | 3.99E-05 | 126 | 115 | 12 | IGKV13-85,IGHV1-53,IGKV2-137,IGHV1-26,IGHV1-66,IGHV3-1,IGKV8-21,IGKV17-121,IGHV3-6,IGLC2,IGHV8-11,IGKV1-110 |
| | REAC | FCGR activation | 3.99E-05 | 126 | 115 | 12 | IGKV13-85,IGHV1-53,IGKV2-137,IGHV1-26,IGHV1-66,IGHV3-1,IGKV8-21,IGKV17-121,IGHV3-6,IGLC2,IGHV8-11,IGKV1-110 |
| | REAC | Creation of C4 and C2 activators | 4.36E-05 | 127 | 115 | 12 | IGKV13-85,IGHV1-53,IGKV2-137,IGHV1-26,IGHV1-66,IGHV3-1,IGKV8-21,IGKV17-121,IGHV3-6,IGLC2,IGHV8-11,IGKV1-110 |
| | GO:BP | sarcomere organization | 5.63E-05 | 45 | 193 | 7 | MYOM2,MYPN,MYOZ1,TCAP,NEB,CASQ1,LDB3 |
| | REAC | Role of phospholipids in phagocytosis | 7.87E-05 | 134 | 115 | 12 | IGKV13-85,IGHV1-53,IGKV2-137,IGHV1-26,IGHV1-66,IGHV3-1,IGKV8-21,IGKV17-121,IGHV3-6,IGLC2,IGHV8-11,IGKV1-110 |
| | REAC | Initial triggering of complement | 7.87E-05 | 134 | 115 | 12 | IGKV13-85,IGHV1-53,IGKV2-137,IGHV1-26,IGHV1-66,IGHV3-1,IGKV8-21,IGKV17-121,IGHV3-6,IGLC2,IGHV8-11,IGKV1-110 |
| | REAC | Antigen activates B Cell Receptor (BCR) leading to generation of second messengers | 8.54E-05 | 135 | 115 | 12 | IGKV13-85,IGHV1-53,IGKV2-137,IGHV1-26,IGHV1-66,IGHV3-1,IGKV8-21,IGKV17-121,IGHV3-6,IGLC2,IGHV8-11,IGKV1-110 |
| | REAC | FCERI mediated Ca+2 mobilization | 0.0001 | 138 | 115 | 12 | IGKV13-85,IGHV1-53,IGKV2-137,IGHV1-26,IGHV1-66,IGHV3-1,IGKV8-21,IGKV17-121,IGHV3-6,IGLC2,IGHV8-11,IGKV1-110 |
| | REAC | FCERI mediated MAPK activation | 0.0001 | 141 | 115 | 12 | IGKV13-85,IGHV1-53,IGKV2-137,IGHV1-26,IGHV1-66,IGHV3-1,IGKV8-21,IGKV17-121,IGHV3-6,IGLC2,IGHV8-11,IGKV1-110 |
| | REAC | Striated Muscle Contraction | 0.0002 | 32 | 140 | 7 | MYBPC1,MYL1,ACTN3,TCAP,NEB,TNNI2,MYBPC2 |
| | GO:BP | skeletal muscle contraction | 0.0003 | 45 | 144 | 6 | TNNT3,ATP2A1,ACTN3,TCAP,TNNI2,CASQ1 |
| | REAC | Regulation of Complement cascade | 0.0003 | 152 | 115 | 12 | IGKV13-85,IGHV1-53,IGKV2-137,IGHV1-26,IGHV1-66,IGHV3-1,IGKV8-21,IGKV17-121,IGHV3-6,IGLC2,IGHV8-11,IGKV1-110 |
| | GO:BP | muscle cell development | 0.0004 | 214 | 144 | 10 | MYOM2,SYPL2,MYPN,MYOZ1,ACTN3,TCAP,NEB,SIX1,ALPK3,CASQ1 |
| | REAC | Complement cascade | 0.0006 | 161 | 115 | 12 | IGKV13-85,IGHV1-53,IGKV2-137,IGHV1-26,IGHV1-66,IGHV3-1,IGKV8-21,IGKV17-121,IGHV3-6,IGLC2,IGHV8-11,IGKV1-110 |
| | REAC | Regulation of actin dynamics for phagocytic cup formation | 0.0009 | 168 | 115 | 12 | IGKV13-85,IGHV1-53,IGKV2-137,IGHV1-26,IGHV1-66,IGHV3-1,IGKV8-21,IGKV17-121,IGHV3-6,IGLC2,IGHV8-11,IGKV1-110 |
| | GO:BP | myofibril assembly | 0.0009 | 67 | 193 | 7 | MYOM2,MYPN,MYOZ1,TCAP,NEB,CASQ1,LDB3 |
| | REAC | Cell surface interactions at the vascular wall | 0.001 | 201 | 115 | 13 | IGKV13-85,IGHV1-53,IGKV2-137,IGHV1-26,IGHV1-66,IGHV3-1,IGKV8-21,IGKV17-121,GRB7,IGHV3-6,IGLC2,IGHV8-11,IGKV1-110 |
| | GO:BP | striated muscle cell development | 0.001 | 68 | 193 | 7 | MYOM2,MYPN,MYOZ1,TCAP,NEB,CASQ1,LDB3 |
| | GO:BP | complement activation | 0.0011 | 177 | 109 | 8 | IGHV1-53,IGHV1-26,IGHV1-66,IGHV3-1,IGHV3-6,KRT1,IGLC2,IGHV8-11 |
| | GO:BP | musculoskeletal movement | 0.0014 | 59 | 144 | 6 | TNNT3,ATP2A1,ACTN3,TCAP,TNNI2,CASQ1 |
| | GO:BP | multicellular organismal movement | 0.0015 | 60 | 144 | 6 | TNNT3,ATP2A1,ACTN3,TCAP,TNNI2,CASQ1 |
| | REAC | Muscle contraction | 0.0023 | 147 | 144 | 12 | TRDN,MYBPC1,MYL1,ATP2A1,ACTN3,MYLPF,TCAP,NEB,KCNJ12,TNNI2,MYBPC2,CASQ1 |
| | REAC | FCERI mediated NF-kB activation | 0.0027 | 187 | 115 | 12 | IGKV13-85,IGHV1-53,IGKV2-137,IGHV1-26,IGHV1-66,IGHV3-1,IGKV8-21,IGKV17-121,IGHV3-6,IGLC2,IGHV8-11,IGKV1-110 |
| | REAC | Fcgamma receptor (FCGR) dependent phagocytosis | 0.0029 | 188 | 115 | 12 | IGKV13-85,IGHV1-53,IGKV2-137,IGHV1-26,IGHV1-66,IGHV3-1,IGKV8-21,IGKV17-121,IGHV3-6,IGLC2,IGHV8-11,IGKV1-110 |
| | GO:BP | cell recognition | 0.0033 | 275 | 109 | 9 | MYOT,MYPN,IGHV1-53,IGHV1-26,IGHV1-66,IGHV3-1,IGHV3-6,IGLC2,IGHV8-11 |
| | GO:BP | phagocytosis, recognition | 0.0048 | 150 | 109 | 7 | IGHV1-53,IGHV1-26,IGHV1-66,IGHV3-1,IGHV3-6,IGLC2,IGHV8-11 |
| | GO:BP | complement activation, classical pathway | 0.006 | 155 | 109 | 7 | IGHV1-53,IGHV1-26,IGHV1-66,IGHV3-1,IGHV3-6,IGLC2,IGHV8-11 |
| | REAC | Signaling by the B Cell Receptor (BCR) | 0.0087 | 210 | 115 | 12 | IGKV13-85,IGHV1-53,IGKV2-137,IGHV1-26,IGHV1-66,IGHV3-1,IGKV8-21,IGKV17-121,IGHV3-6,IGLC2,IGHV8-11,IGKV1-110 |

## Slide 24
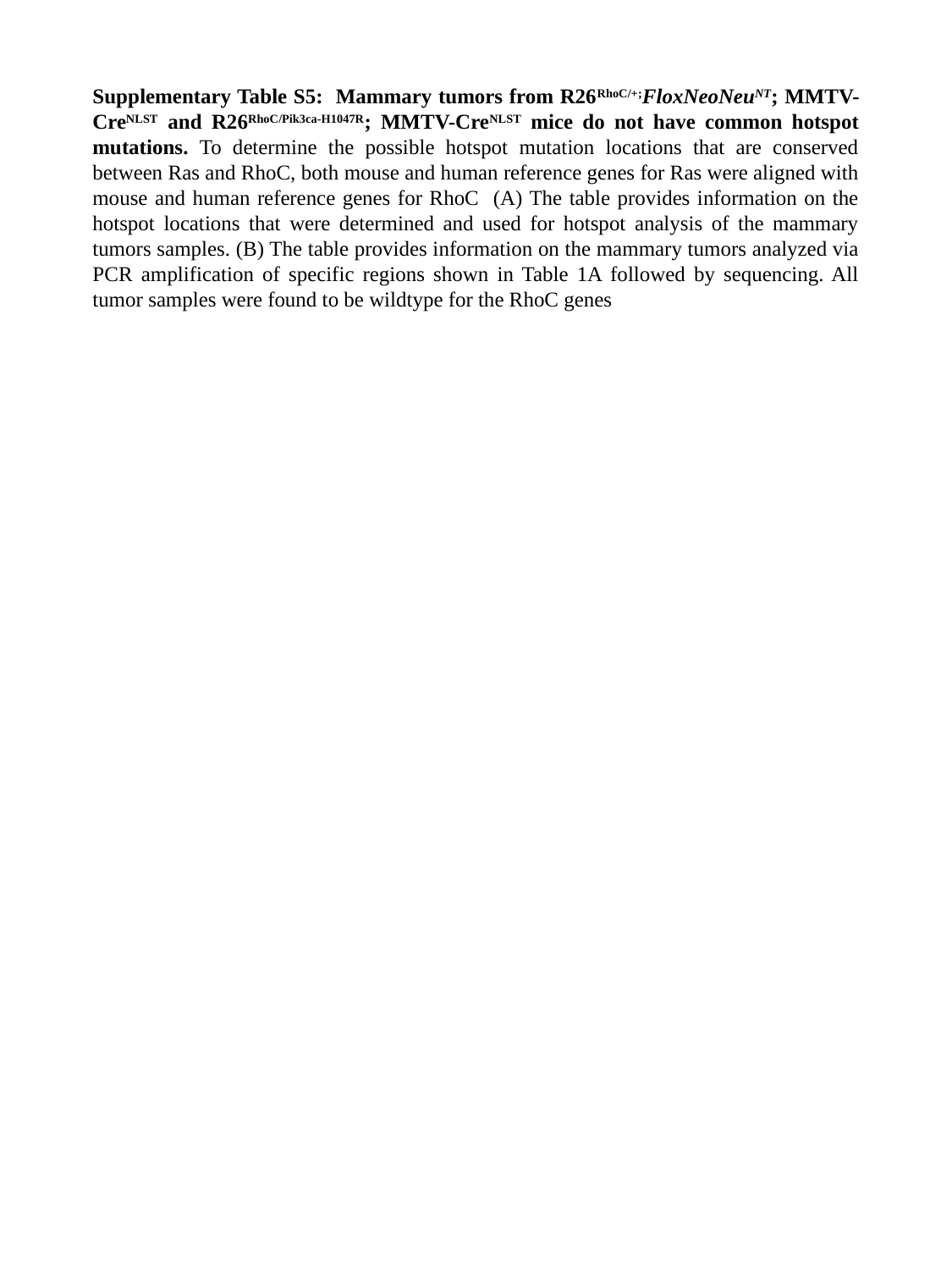

Supplementary Table S5: Mammary tumors from R26RhoC/+;FloxNeoNeuNT; MMTV-CreNLST and R26RhoC/Pik3ca-H1047R; MMTV-CreNLST mice do not have common hotspot mutations. To determine the possible hotspot mutation locations that are conserved between Ras and RhoC, both mouse and human reference genes for Ras were aligned with mouse and human reference genes for RhoC (A) The table provides information on the hotspot locations that were determined and used for hotspot analysis of the mammary tumors samples. (B) The table provides information on the mammary tumors analyzed via PCR amplification of specific regions shown in Table 1A followed by sequencing. All tumor samples were found to be wildtype for the RhoC genes

## Slide 25
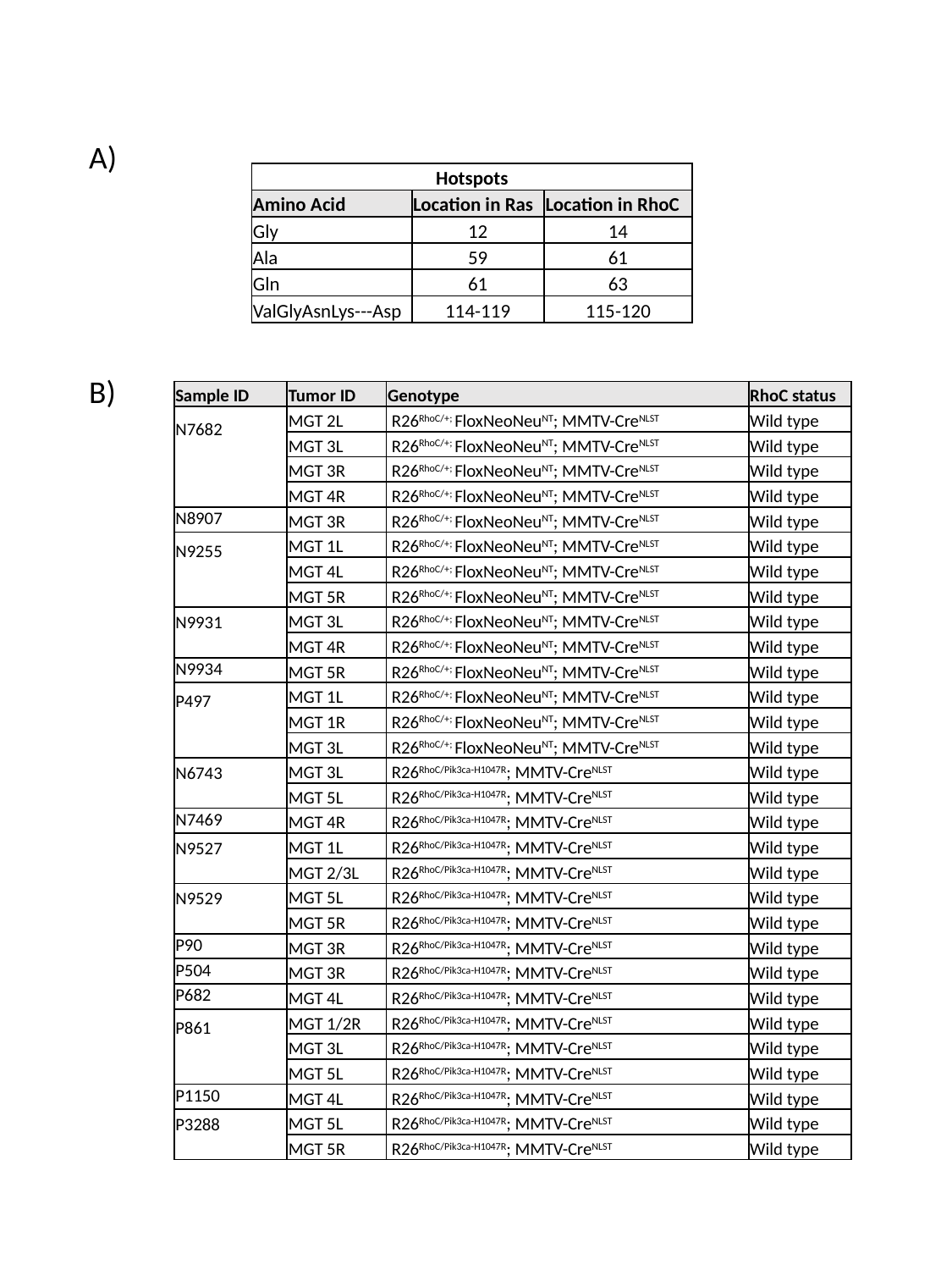

A)
| Hotspots | | |
| --- | --- | --- |
| Amino Acid | Location in Ras | Location in RhoC |
| Gly | 12 | 14 |
| Ala | 59 | 61 |
| Gln | 61 | 63 |
| ValGlyAsnLys---Asp | 114-119 | 115-120 |
B)
| Sample ID | Tumor ID | Genotype | RhoC status |
| --- | --- | --- | --- |
| N7682 | MGT 2L | R26RhoC/+; FloxNeoNeuNT; MMTV-CreNLST | Wild type |
| | MGT 3L | R26RhoC/+; FloxNeoNeuNT; MMTV-CreNLST | Wild type |
| | MGT 3R | R26RhoC/+; FloxNeoNeuNT; MMTV-CreNLST | Wild type |
| | MGT 4R | R26RhoC/+; FloxNeoNeuNT; MMTV-CreNLST | Wild type |
| N8907 | MGT 3R | R26RhoC/+; FloxNeoNeuNT; MMTV-CreNLST | Wild type |
| N9255 | MGT 1L | R26RhoC/+; FloxNeoNeuNT; MMTV-CreNLST | Wild type |
| | MGT 4L | R26RhoC/+; FloxNeoNeuNT; MMTV-CreNLST | Wild type |
| | MGT 5R | R26RhoC/+; FloxNeoNeuNT; MMTV-CreNLST | Wild type |
| N9931 | MGT 3L | R26RhoC/+; FloxNeoNeuNT; MMTV-CreNLST | Wild type |
| | MGT 4R | R26RhoC/+; FloxNeoNeuNT; MMTV-CreNLST | Wild type |
| N9934 | MGT 5R | R26RhoC/+; FloxNeoNeuNT; MMTV-CreNLST | Wild type |
| P497 | MGT 1L | R26RhoC/+; FloxNeoNeuNT; MMTV-CreNLST | Wild type |
| | MGT 1R | R26RhoC/+; FloxNeoNeuNT; MMTV-CreNLST | Wild type |
| | MGT 3L | R26RhoC/+; FloxNeoNeuNT; MMTV-CreNLST | Wild type |
| N6743 | MGT 3L | R26RhoC/Pik3ca-H1047R; MMTV-CreNLST | Wild type |
| | MGT 5L | R26RhoC/Pik3ca-H1047R; MMTV-CreNLST | Wild type |
| N7469 | MGT 4R | R26RhoC/Pik3ca-H1047R; MMTV-CreNLST | Wild type |
| N9527 | MGT 1L | R26RhoC/Pik3ca-H1047R; MMTV-CreNLST | Wild type |
| | MGT 2/3L | R26RhoC/Pik3ca-H1047R; MMTV-CreNLST | Wild type |
| N9529 | MGT 5L | R26RhoC/Pik3ca-H1047R; MMTV-CreNLST | Wild type |
| | MGT 5R | R26RhoC/Pik3ca-H1047R; MMTV-CreNLST | Wild type |
| P90 | MGT 3R | R26RhoC/Pik3ca-H1047R; MMTV-CreNLST | Wild type |
| P504 | MGT 3R | R26RhoC/Pik3ca-H1047R; MMTV-CreNLST | Wild type |
| P682 | MGT 4L | R26RhoC/Pik3ca-H1047R; MMTV-CreNLST | Wild type |
| P861 | MGT 1/2R | R26RhoC/Pik3ca-H1047R; MMTV-CreNLST | Wild type |
| | MGT 3L | R26RhoC/Pik3ca-H1047R; MMTV-CreNLST | Wild type |
| | MGT 5L | R26RhoC/Pik3ca-H1047R; MMTV-CreNLST | Wild type |
| P1150 | MGT 4L | R26RhoC/Pik3ca-H1047R; MMTV-CreNLST | Wild type |
| P3288 | MGT 5L | R26RhoC/Pik3ca-H1047R; MMTV-CreNLST | Wild type |
| | MGT 5R | R26RhoC/Pik3ca-H1047R; MMTV-CreNLST | Wild type |

## Slide 26
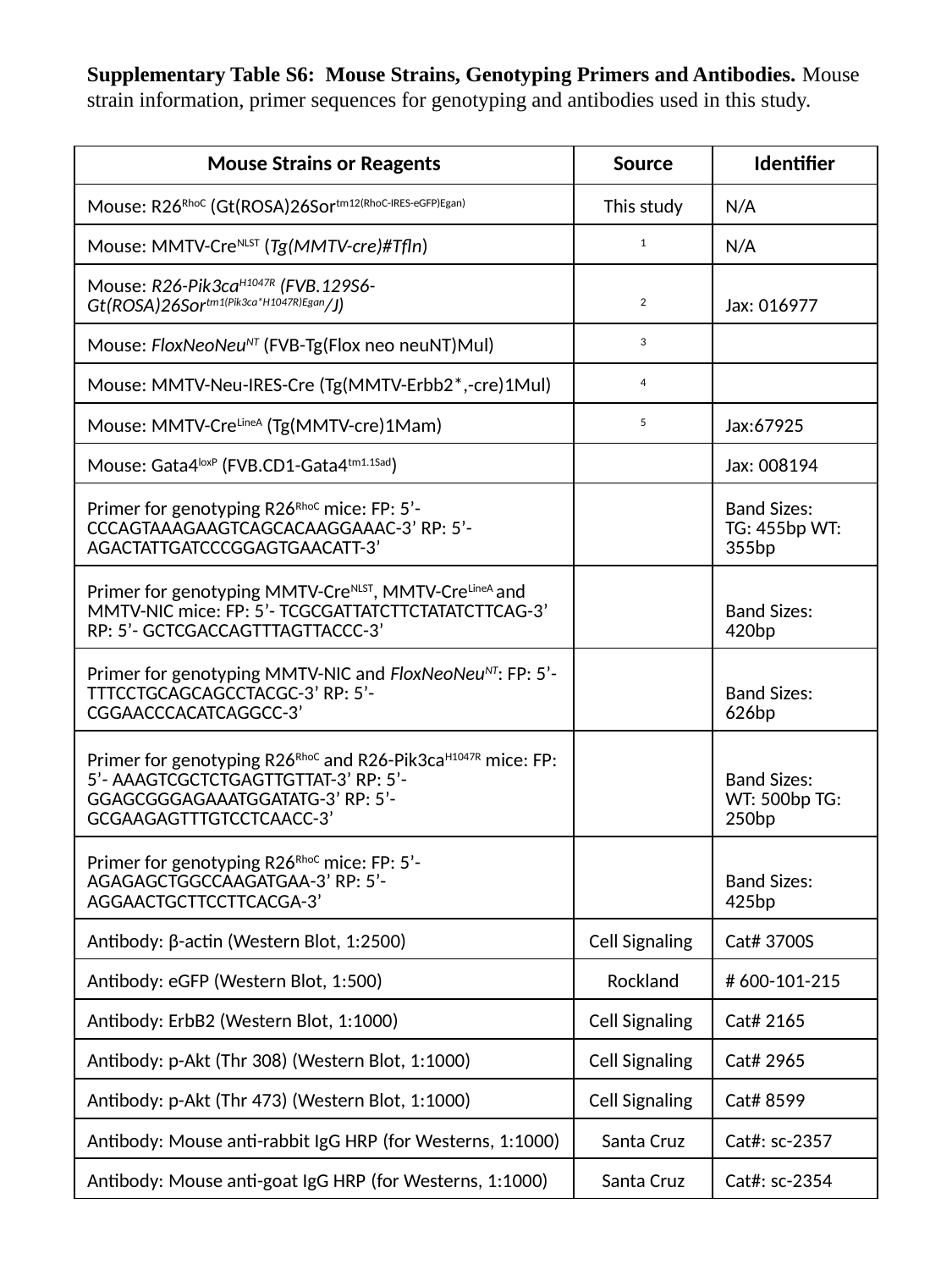

Supplementary Table S6: Mouse Strains, Genotyping Primers and Antibodies. Mouse strain information, primer sequences for genotyping and antibodies used in this study.
| Mouse Strains or Reagents | Source | Identifier |
| --- | --- | --- |
| Mouse: R26RhoC (Gt(ROSA)26Sortm12(RhoC-IRES-eGFP)Egan) | This study | N/A |
| Mouse: MMTV-CreNLST (Tg(MMTV-cre)#Tfln) | 1 | N/A |
| Mouse: R26-Pik3caH1047R (FVB.129S6- Gt(ROSA)26Sortm1(Pik3ca\*H1047R)Egan/J) | 2 | Jax: 016977 |
| Mouse: FloxNeoNeuNT (FVB-Tg(Flox neo neuNT)Mul) | 3 | |
| Mouse: MMTV-Neu-IRES-Cre (Tg(MMTV-Erbb2\*,-cre)1Mul) | 4 | |
| Mouse: MMTV-CreLineA (Tg(MMTV-cre)1Mam) | 5 | Jax:67925 |
| Mouse: Gata4loxP (FVB.CD1-Gata4tm1.1Sad) | | Jax: 008194 |
| Primer for genotyping R26RhoC mice: FP: 5’- CCCAGTAAAGAAGTCAGCACAAGGAAAC-3’ RP: 5’- AGACTATTGATCCCGGAGTGAACATT-3’ | | Band Sizes: TG: 455bp WT: 355bp |
| Primer for genotyping MMTV-CreNLST, MMTV-CreLineA and MMTV-NIC mice: FP: 5’- TCGCGATTATCTTCTATATCTTCAG-3’ RP: 5’- GCTCGACCAGTTTAGTTACCC-3’ | | Band Sizes: 420bp |
| Primer for genotyping MMTV-NIC and FloxNeoNeuNT: FP: 5’- TTTCCTGCAGCAGCCTACGC-3’ RP: 5’- CGGAACCCACATCAGGCC-3’ | | Band Sizes: 626bp |
| Primer for genotyping R26RhoC and R26-Pik3caH1047R mice: FP: 5’- AAAGTCGCTCTGAGTTGTTAT-3’ RP: 5’- GGAGCGGGAGAAATGGATATG-3’ RP: 5’- GCGAAGAGTTTGTCCTCAACC-3’ | | Band Sizes: WT: 500bp TG: 250bp |
| Primer for genotyping R26RhoC mice: FP: 5’- AGAGAGCTGGCCAAGATGAA-3’ RP: 5’- AGGAACTGCTTCCTTCACGA-3’ | | Band Sizes: 425bp |
| Antibody: β-actin (Western Blot, 1:2500) | Cell Signaling | Cat# 3700S |
| Antibody: eGFP (Western Blot, 1:500) | Rockland | # 600-101-215 |
| Antibody: ErbB2 (Western Blot, 1:1000) | Cell Signaling | Cat# 2165 |
| Antibody: p-Akt (Thr 308) (Western Blot, 1:1000) | Cell Signaling | Cat# 2965 |
| Antibody: p-Akt (Thr 473) (Western Blot, 1:1000) | Cell Signaling | Cat# 8599 |
| Antibody: Mouse anti-rabbit IgG HRP (for Westerns, 1:1000) | Santa Cruz | Cat#: sc-2357 |
| Antibody: Mouse anti-goat IgG HRP (for Westerns, 1:1000) | Santa Cruz | Cat#: sc-2354 |

## Slide 27
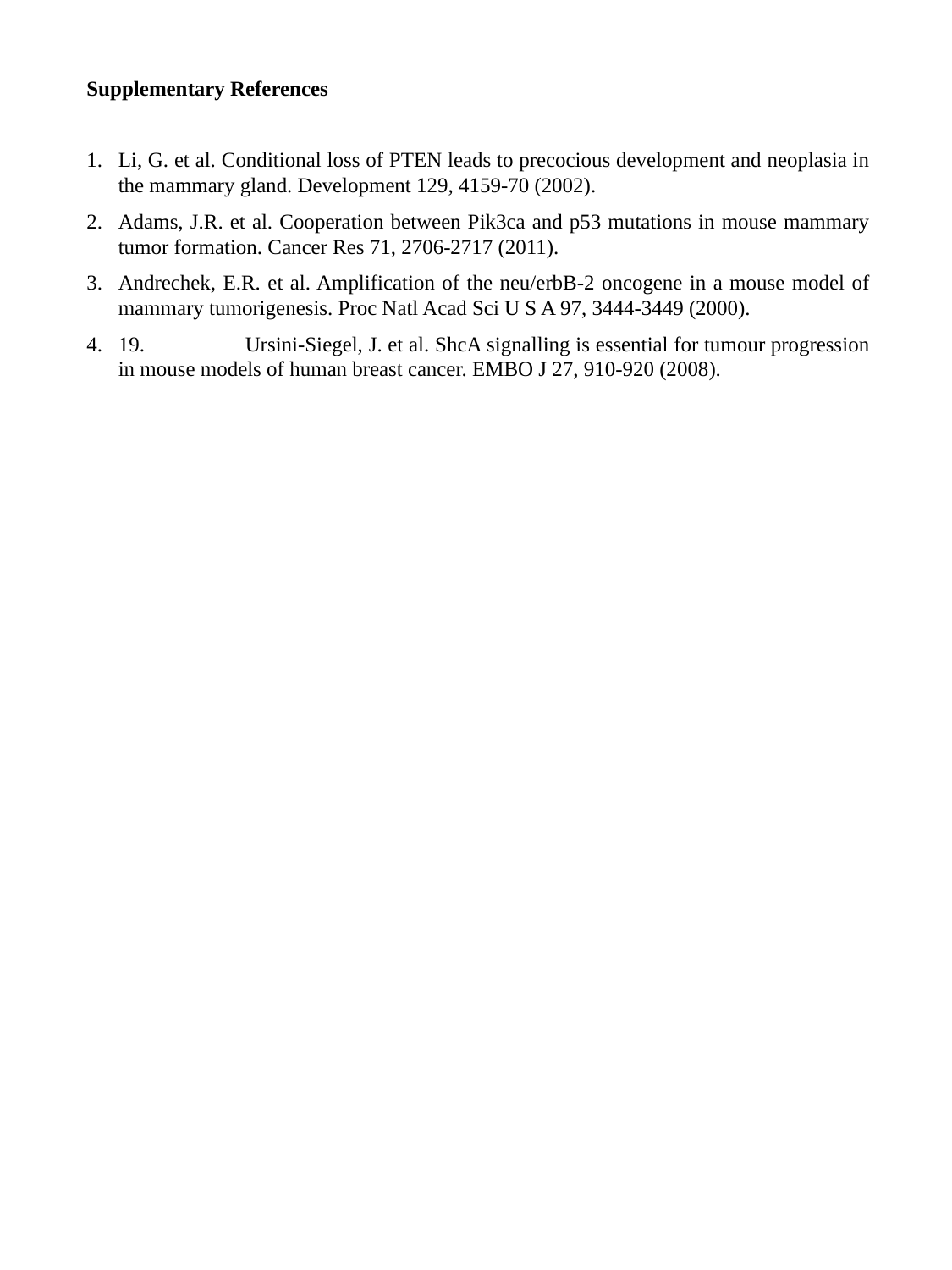

Supplementary References
Li, G. et al. Conditional loss of PTEN leads to precocious development and neoplasia in the mammary gland. Development 129, 4159-70 (2002).
Adams, J.R. et al. Cooperation between Pik3ca and p53 mutations in mouse mammary tumor formation. Cancer Res 71, 2706-2717 (2011).
Andrechek, E.R. et al. Amplification of the neu/erbB-2 oncogene in a mouse model of mammary tumorigenesis. Proc Natl Acad Sci U S A 97, 3444-3449 (2000).
19.	Ursini-Siegel, J. et al. ShcA signalling is essential for tumour progression in mouse models of human breast cancer. EMBO J 27, 910-920 (2008).
